# Decoration of gold nanoparticles with glycopeptides leads to a lower cellular uptake and liver retention†

**DOI:** 10.1039/d5na00464k

**Published:** 2025-08-12

**Authors:** Mahmoud G. Soliman, Jennifer Fernandez Alarcon, Tanja Ursula Lüdtke, Martina B. Violatto, Marko Dobricic, Chiara Cordiglieri, Alessandro Corbelli, Fabio Fiordaliso, Giovanni Sitia, James S. O'Donnell, Daniel I. R. Spencer, Sergio Moya, Paolo Bigini, Marco P. Monopoli

**Affiliations:** a Department of Chemistry, Royal College of Surgeons of Ireland (RCSI) St Stephens Green 123 Dublin Ireland marcomonopoli@rcsi.ie; b Physics Department, Faculty of Science, Al-Azhar University Cairo Egypt; c Department of Molecular Biochemistry and Pharmacology, Istituto di Ricerche Farmacologiche Mario Negri IRCCS Via Mario Negri 2 20156 Milano Italy paolo.bigini@marionegri.it; d Department of Soft Matter Nanotechnology, CIC Biomagune Paseo Miramon 182 20014 San Sebastian-Donostia Spain; e INGM Imaging Facility, Istituto Nazionale Genetica Molecolare Via Francesco Sforza 35 20122 Milano Italy; f Experimental Hepatology Unit, Division of Immunology, Transplantation and Infectious Diseases, IRCCS San Raffaele Scientific Institute Via Olgettina 58 20132 Milano Italy; g Irish Centre for Vascular Biology, School of Pharmacy and Biomolecular Sciences, Royal College of Surgeons in Ireland (RCSI) Dublin Ireland; h Ludger Ltd, Culham Science Centre Abingdon Oxfordshire OX14 3EB UK

## Abstract

Unspecific uptake by the liver is one of the main drawbacks of the translation of nanomaterials into clinics, preventing their delivery into diseased tissues. Here, we synthesized gold nanoparticles (GNPs) decorated with a sialic acid-displaying glycopeptide to enhance their specific targeting properties by reducing their uptake inside hepatic cells. We demonstrated the biocompatibility of the glycopeptide-coated GNPs with two different nanomaterial shapes (spherical and rod-like GNPs) and the targeting properties of the glycopeptide were retained in serum-free and protein-rich media. We found that the glycopeptide reduces nanomaterial interaction with hepatic cells by 1.96 times. In the liver, Kupffer cells (KCs) and liver sinusoidal endothelial cells (LSECs) were the only cells that interacted with the GNPs, increasing the expression of sialic acid-binding receptors such as Siglec-1. This work provides potential new strategies to overcome off-target nanomaterial accumulation by manipulating nanomaterial functionalisation with glycans to alter hepatic cell interactions.

## Abstract 2

Gold nanoparticles decorated with a sialic acid-displaying glycopeptide exhibit reduced cellular uptake and liver accumulation, offering a promising strategy to enhance the safety and therapeutic performance of nanomaterials.

## Graphical abstract

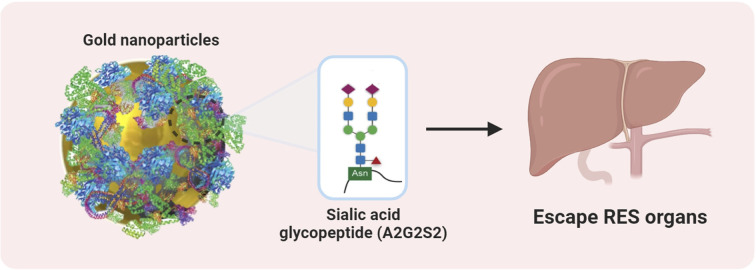

## 1. Introduction

Owing to their unique physico-chemical properties, gold nanoparticles (GNPs) are being tested in preclinical studies for several medical applications, including photo-thermal therapy,^1–3^ drug delivery,^3^ and bioimaging.^4^ However, the GNPs' translation into the clinic has generally been impaired by their fast accumulation into organs from the reticuloendothelial system (RES), such as the liver and spleen, through the mononuclear phagocyte system (MPS).^5–7^ Once trapped in the liver, GNPs have been reported to interact with several hepatic cells, including hepatocytes, liver sinusoidal endothelial cells (LSECs), B cells, and Kupffer cells (KCs). Specifically, GNPs' interaction with KCs leads to hepatic accumulation, as KCs are known to phagocytose foreign particles as a mechanism of defence.^8^ However, other factors like particle size, shape, and surface chemistry have been reported to contribute to the accumulation of GNPs in the liver. In a long-term *in vivo* study, a high hepatic accumulation of uncoated GNPs was observed within 1 min of injection with more than 70% of the smallest nanoparticle (NP) size accumulating.^9^ Other studies have demonstrated that different GNP core volumes, in combination with distinct surface coatings, result in different hepatic accumulation rates.^10^ Additionally, the adsorption of proteins on the NP surface, known as the protein corona, plays a key role in the NPs' unspecific interactions with hepatic cells. It has been reported that the proteins present in the corona can mask the targeting moieties displayed on the NPs,^11^ interfering with their recognition capability and thus facilitating NP uptake by hepatic immune cells and consequent elimination in the liver.^12,13^ Therefore, developing NPs capable of escaping liver retention can be key to enhancing nanomaterials use in clinics for medical applications.

For this reason, many efforts have been devoted to prolonging NP circulation half-life in the bloodstream by exploiting the use of synthetic polymers such as polyethylene glycol (PEG) to functionalize the NP surface and escape MPS clearance.^11^ PEG has been widely used due to its excellent properties, like increased colloidal stability, less undesired interactions with proteins or other blood components, and a longer blood circulation lifetime.^14–17^ However, this polymer, apart from provoking immune reactions in humans and other animals,^18^ can mitigate drug release and hide targeting moieties, thereby compromising the therapeutic goals.^19,20^ Therefore, there is an unmet need to develop new strategies capable of replacing PEG or enhancing NP stealthiness without compromising their targeting capability in the circulatory system.

Glycans have emerged as an alternative to PEG, with exploitable properties such as antifouling and biological recognition, making them unique candidates for use in the field of nanomedicine.^21–23^ Glycans are naturally occurring biomolecules made of sugar units that can selectively bind to relevant proteins to form glycoproteins, and therefore, play an important role in many key biological processes.^24^ An example of glycan-linked proteins is the biantennary digalactosylated α2,6 linked disialylated (A2G2S2) oligosaccharide, one of the most abundant proteins found in human plasma.^25^ Sialic acid (Sia) is the most common monosaccharide found in glycans, typically located at the terminal position of glycan chains, and usually linked to galactose residues through either an α2–3 or α2–6 linkage. Sia regulates cellular and molecular interactions by selectively binding to sialic acid-binding immunoglobulin-like lectins (Siglecs) expressed by immune cells, either masking recognition sites or serving as recognition determinants.^26–29^ It plays a pivotal role in determining the half-life of glycoproteins in blood circulation. The lack of Sias could lead to rapid clearance of the protein from circulation as a consequence of binding to the asialoglycoprotein receptor (ASGP), present in the liver and other organs.^26,28,30^ Thus, NP surface decoration with A2G2S2 may hide specific recognition sites from hepatic immune cells, resulting in prolonged NP circulation in the bloodstream and thus reducing the nanomaterials uptake in hepatic cells.

Here, we report the use of glycopeptides, biantennary digalactosylated α2,6-linked disialylated glycan (A2G2S2), as a surface coating for two GNP cores with different aspect ratios (spherical and rod-like NPs). Unlike silicic acid, which has been studied as a single molecule for its ability to reduce macrophage uptake and enhance targeting efficiency,^22^ the A2G2S2 glycopeptide used in this study features a unique and branched structure incorporating two sialic acid residues. This glycopeptide is attached to the asparagine amino acid of a peptide with the sequence lysine–valine–alanine–asparagine–lysine–threonine. The use of this glycosylated peptide ensures correct glycan orientation after the conjugation process, and the α2,6 linkage ensures high selectivity towards Siglec receptors, where the orientation of sialic acid is known to modulate specificity towards the receptors. This interaction modulates self-recognition, helping to suppress inflammation and enabling evasion of phagocytosis.^57^ The targeting properties of A2G2S2 conjugated NPs were tested in a complex biological medium. Additionally, the A2G2S2-conjugated GNPs have been evaluated for their biological response both *in vitro* and *in vivo*. *In vitro* studies using HepG2 showed that A2G2S2 conjugated NPs did not show toxicity (up to 200 μg per mL NPs) and retained their targeting properties in serum-free and protein-rich media. Additionally, the A2G2S2 conjugated GNPs showed lower cellular uptake compared to non-glycosylated GNPs. Similar results were observed *in vivo*, where the ability to avoid the uptake by RES organs was exclusive to rod-like GNPs (GNRs); 97% of non-glycosylated GNRs were rapidly internalised in the liver; the presence of A2G2S2 reduced the hepatic accumulation. The safety of A2G2S2 conjugated GNPs was confirmed by the absence of hepatic immune cell infiltrations and standard enzymatic levels. Taken together, these results suggest that A2G2S2 coating, combined with particle core volume, is an effective strategy to enhance their targeting properties in protein-rich media, revealing their potential as selective drug delivery systems.

## 2. Materials and methods

All glassware was washed with aqua regia and rinsed thoroughly with Milli-Q water before use. All chemicals were used as received. Hydrogen tetrachloroaurate(iii) hydrate (HAuCl_4_), hexadecyltrimethylammonium bromide (CTAB), hexadecyltrimethylammonium chloride (CTAC), sodium borohydride (NaBH_4_), ascorbic acid (AA), ethyl-3-(3-dimethylaminopropyl)carbodiimide (EDC), sulfo-*N*-hydroxysuccinimide (sulfo-NHS), sodium hypochlorite solution, dodecylamine (DDA), poly(isobutylene-*alt*-maleic-anhydride), hydrochloric acid, 5-bromosalicylic acid, silver nitrate (AgNO_3_), phosphate-buffered saline (PBS), Tris–Cl, ethylenediaminetetraacetic acid (EDTA), α-methoxy-ω-thiol-poly(ethylene glycol) (MPEG-SH, *M*_w_ = 2 kDa (PEG 2k) were purchased from Sigma-Aldrich (UK). Biantennary digalactosylated α2,6-linked disialylated glycan (A2G2S2) was purchased from FUJIFILM Wako Chemicals Europe GmbH. Sambucus Nigra Lectin (SNA) was purchased from Vector Laboratories. Dulbecco's Modified Eagle Medium (DMEM), fetal bovine serum (FBS), penicillin, streptomycin, and trypsin were purchased from Thermo Fisher Scientific. All *in vitro* and *in vivo* procedures were performed in compliance with relevant laws and institutional guidelines and have been approved by the appropriate institutional committees. Human blood plasma (hBs) was obtained from the Irish Blood Transfusion Service (IBTS), St. James' Hospital, Dublin. Access to and use of plasma samples were covered by RCSI ethics number 001246b. Most Buffers were prepared manually using Milli-Q water (resistivity of 18.2 MΩ cm at *ca.* 25 °C). The *in vivo* work was covered under the associated code 9F5F5.176, project number 49/2021-PR.

### 2.1. Synthesis of spherical gold nanoparticles (GNPs)

Spherical GNPs with nominal core diameters of *ca.* 55 nm, named 55-GNPs, were synthesized through a three-step synthesis method following a modified protocol reported by Hanske, *et al.*^31^ Step 1: preparation of Au seeds (∼4 nm): to synthesize Au seeds, 200 μL of freshly prepared 0.02 M NaBH_4_ was injected into a mixture of 5 mL of 0.1 M CTAC and 50 μL of 0.05 M HAuCl_4_. This mixture was stirred vigorously for 3 minutes. The resulting seed solution was then diluted 10 times in 100 mM CTAC for use in the subsequent step. Step 2: growth of 10 nm Au nanospheres: the 4 nm Au seeds were grown into 10 nm spheres by adding 50 μL of 0.05 M HAuCl_4_ to a mixture containing 0.9 mL of the seed solution, 0.04 mL of 0.1 M AA, and 10 mL of 25 mM CTAC. After 10 seconds, stirring was stopped, and the mixture was left at room temperature for at least 10 minutes. The resulting gold nanospheres exhibited a localized surface plasmon resonance (LSPR) band centred at 521 nm. Step 3: growth of 55 nm Au nanospheres. For large-scale production of 55 nm spheres, the growth protocol was modified from Christoph's method. A mixture of 500 mL of 25 mM CTAC and 2 mL of 0.1 M AA was prepared under gentle stirring. Then, 1.850 mL of the 10 nm spheres was injected. Following this, 2.5 mL of 0.05 M HAuCl_4_ was added while stirring vigorously. The mixture was left undisturbed at room temperature for at least 1 hour. To achieve a perfect spherical shape, 1 mL of sodium hypochlorite solution (1 to 1.5 wt% available chlorine) was added under vigorous stirring. After 5 minutes of stirring, 0.125 mL of 0.05 M HAuCl_4_ was injected. The particle solution was then incubated at 37 °C for at least 2 h to ensure the completion of the oxidative etching process. After synthesis, the GNP solution was purified by centrifugation (refer to Table S1† for specific centrifugation conditions). Then, the supernatant was discarded, and the pellet was re-dispersed in Milli-Q water for storage and further use.

### 2.2. Synthesis of gold nanorods (GNRs)

GNRs with dimensions of *ca.*16 × 65 nm, named 65-GNRs, were prepared following the protocol reported by Ye *et al.*^32^ Step 1: synthesis of 4 nm Au seeds. To prepare the Au seeds, 0.6 mL of a 0.02 M NaBH_4_ solution, pre-diluted in 1 mL of Milli-Q water, was injected into a vigorously stirred solution containing 5 mL of 0.2 M CTAB and 5 mL of 0.5 mM HAuCl_4_. The resulting brown-yellow solution was allowed to sit at room temperature for 15 minutes before use. Step 2: preparation of the growth solution. A growth solution was created by dissolving 9.0 g of CTAB and 1.1 g of 5-bromosalicylic acid in 250 mL of warm water. After the solution cooled to 30 °C, 18 mL of 4 mM AgNO_3_ was added. This mixture was maintained at 30 °C for 15 minutes, after which 250 mL of 1 mM HAuCl_4_ was introduced. Following 15 minutes of stirring at 400 rpm, 2 mL of 0.064 M AA was injected, and the solution was vigorously stirred for 30 seconds until it became colorless. Next, 0.4 mL of the freshly prepared seed solution (from Step 1) was injected into the growth solution and stirred for an additional 30 seconds. The solution was then allowed to sit overnight at 30 °C without stirring to promote GNR growth. The resulting GNR solution was cleaned through centrifugation (see Table S1† for specific centrifugation conditions). The supernatant was discarded, and the pellet was re-dispersed in Milli-Q water for storage and future use.

### 2.3. Nanoparticle phase transfer and polymer coating

The polymer coating technique for NPs involved transferring them from an aqueous solution to an organic solvent, as detailed by Soliman *et al.*^33^ To begin, a small volume of PEG 2k in water (see Table S1†) was introduced into the NP solution to help stabilize the particles when they came into contact with the organic solvent. Phase transfer was achieved by mixing the NPs with 60 mmol of DDA in an equal volume of chloroform. The mixture was stirred until the NPs successfully transferred into the chloroform phase. Following this, the particles were subjected to two rounds of centrifugation (refer to Table S1†) to remove any unbound organic compounds (DDA and PEG 2k). The resulting pellet was then redispersed in chloroform for future use.

In the next phase, the DDA-capped NPs were retransferred to an aqueous medium using the polymer coating approach described by Lin *et al.*^34^ The amphiphilic polymer, poly(isobutylene-*alt*-maleic anhydride), was synthesized by attaching DDA to its hydrophilic backbone through spontaneous amide bond formation. This reaction transforms one maleic anhydride unit into an amide and a free carboxylic acid. Seventy-five percent of the polymer backbone was functionalized to create a hydrophobic section, enabling it to integrate with the hydrophobic surfactant layer on the NPs. The remaining 25% of unopened anhydride rings facilitated the transfer of the NPs into water, providing stability through negatively charged carboxyl groups. For a comprehensive overview of the polymer synthesis, refer to Lin *et al.*^34^ The resultant dodecyl-grafted poly(isobutylene-*alt*-maleic anhydride), henceforth called PMA, was prepared as a 0.5 M stock solution in chloroform. The amount of PMA to be added per NP was determined based on the calculations presented by Xu and colleagues.^10^ The coating process was then executed using the previously mentioned protocol.^33^

### 2.4. Glycopeptide conjugation

The negatively charged carboxylic acid groups (–COOH) on the PMA-coated NPs were utilized to link the glycopeptides through an EDC/sulfo-NHS coupling reaction.^35^ Briefly, 0.5 μmol of EDC and 1 μmol of sulfo-NHS were added to 100 μg of NP suspension (55-GNPs or 65-GNRs) in 10 mM SBB9. The mixture was left undisturbed for 20 min at RT, followed by injecting 75 μg of A2G2S2 and was then left overnight. Excess unreacted chemicals were removed by centrifugation using the same parameters described in Table S1.† The resulting A2G2S2 conjugated PMA_55-GNPs/65-GNRs (hereafter referred to as A2G2S2_55-GNPs/65-GNRs) were resuspended in Milli-Q water and stored at RT for further use.

### 2.5. Lectin binding assay in buffer

For this study, a stock solution of SNA (1 mg mL^−1^) was prepared in PBS and stored at −20 °C for further use. A2G2S2_55-GNPs/65-GNRs (5 μg) were dispersed in PBS (0.1 mL), followed by the addition of SNA (10 μg). The mixtures were then incubated for 1 h without disturbance. A positive control of A2G2S2_55-GNPs/65-GNRs was incubated under the same conditions in the absence of SNA. To study the affinity of SNA towards non-glycosylated PMA_55-GNPs/65-GNRs, non-glycosylated PMA-coated NPs were incubated in the presence and absence of SNA under the same incubation conditions. After that, the absorbance spectra and the hydrodynamic size were recorded by UV-vis spectroscopy and dynamic light scattering (DLS), respectively.

### 2.6. Protein corona preparation and lectin binding assay in protein-rich media

On the day of the experiment, BSA solutions were freshly prepared at concentrations of 30 mg mL^−1^ and 60 mg mL^−1^. FBS was also thawed and diluted in PBS to prepare 10% and 80% volume/volume (v/v) solutions. To assess the formation of the hard corona, PMA_55-GNPs/65-GNRs (50 μg) were incubated with BSA or FBS (10% and 80% concentrations). These mixtures were incubated for 1 h at 37 °C with shaking at 300 rpm. Following incubation, any unbound biomolecules were removed through centrifugation as previously described. The resulting NP-hard corona (NP-HC) complexes were collected in 20 μL of PBS for further characterization.

For competitive binding assays in a protein-rich environment, A2G2S2-functionalized 55-GNPs/65-GNRs (50 μg) were added to freshly prepared BSA solutions at concentrations of 30 mg mL^−1^ or 60 mg mL^−1^ in PBS. Subsequently, SNA (10 μg) was introduced, and the mixtures were incubated undisturbed for 24 h. Control samples, including non-glycosylated PAM_55-GNPs/65-GNRs and A2G2S2_55-GNPs/65-GNRs, were incubated under the same conditions without the addition of SNA. After the incubation period, unbound biomolecules were removed *via* centrifugation, and the resulting nanoparticles were then used for characterization.

### 2.7. Physicochemical characterization

UV-vis absorption spectra of the nanoparticles (NPs) with various surface coatings were recorded across a wavelength range of 400–1000 nm using a UV/vis spectrophotometer (Agilent 8453). For spherical GNPs, a characteristic absorption peak in the visible region, known as the surface plasmon resonance (SPR), was observed. In contrast, for GNRs, the SPR band was split into two distinct peaks: a weaker band in the visible region (which is largely unaffected by size changes) and a stronger band in the near-infrared (NIR) region. The optical properties of both spherical GNPs and GNRs were analyzed by monitoring the shifts and intensity changes in the SPR peaks—located in the visible region for GNPs and in the NIR region for GNRs. The measurements were conducted in either water or PBS using a plastic cuvette with a path length of 1 cm. The molar concentration of the NPs was calculated using the Beer–Lambert Law, with UV/vis absorbance measurements taken at 450 nm (*A*_450_). The extinction coefficients (*ε*) at 450 nm were 1.26 × 10^10^ [M^−1^ cm^−1^] for 55 nm GNPs and 2.11 × 10^9^ [M^−1^ cm^−1^] for 65 nm GNRs.^36,37^

Dynamic light scattering (DLS) and laser Doppler anemometry (LDA) were employed to measure the hydrodynamic diameter (*d*_h_) and zeta potential (ζ-potential) of the GNPs in water or PBS, using a Nanosizer from Malvern. Before measurement, all samples were equilibrated for 5 minutes at 25 °C to ensure that the nanoparticle motion resulted solely from Brownian motion, eliminating any influence from thermal gradients. The measurements were conducted at a backscatter angle of 173°, utilizing a 633 nm laser. The resulting *d*_h_ values, reported in nanometers (nm), and the ζ-potential values, expressed in millivolts (mV), are summarized in Table S2.† Each value presented is the average of at least three independent measurements to ensure accuracy and reliability.

The quality of the NPs was characterized using a transmission electron microscope (TEM, Tecnai G3 Spirit). For sample preparation, a 5 μL drop of diluted NP solution was placed on a carbon-coated copper grid and allowed to air dry at RT. TEM images were then captured and analyzed to calculate the size distribution of the NPs. Image analysis was performed using free ImageJ software, and the average NP size was determined by measuring the cores of at least 100 individual NPs.

Surface modification of the NPs was assessed through gel electrophoresis using a Wide Mini-Sub Cell GT system (Bio-Rad). A 1% agarose gel was prepared in Tris–EDTA buffer (pH 7.4) for the experiment. Before loading, the NP solutions were mixed with the gel-loading buffer, “orange G,” which ensured the stability of the NPs and helped them settle at the bottom of the wells. The electrophoresis was conducted under an electric field of 100 V at RT for 1 hour in Tris–EDTA buffer using a Bio-Rad horizontal electrophoresis setup. Afterward, the gel image was captured for analysis.

Sodium dodecyl sulfate polyacrylamide gel electrophoresis (SDS-PAGE) was employed to analyze the proteins adsorbed on the surface of the NPs after their exposure to hBs, FBS, or BSA (refer to the above protocol for biomolecular corona preparation and purification). For this analysis, 12 μL of GNPs were mixed with 6 μL of SDS-PAGE loading buffer containing 187.5 mM Tris–HCl (pH 6.8), 6% (w/v) SDS, 30% (v/v) glycerol, 0.15 M DTT, 2% β-mercaptoethanol, and 0.03% (w/v) bromophenol blue. The mixture was incubated at 90 °C for 10 minutes to desorb the proteins from the NP surface. The resulting protein samples were then separated on a 10% SDS-PAGE gel using a MiniPROTEAN3 system (Bio-Rad Inc.). Following electrophoresis, the gels were stained using the Coomassie brilliant blue method and subsequently destained until areas of gelatinolytic or caseinolytic activity appeared as clear bands. The final gel, showing sharp bands against a dark blue background, was imaged using a Bio-Rad ChemiDoc™ MP Imaging System (Bio-Rad Inc.).

### 2.8. LAL assay for gold NPs

To evaluate potential endotoxin (lipopolysaccharide, LPS) contamination in the NP solutions during production or handling, the samples were analyzed using a Pierce LAL Chromogenic Endotoxin Quantitation Kit (88282) from ThermoFisher. In brief, an aliquot of the NP stock, equivalent to the amount administered to mice, was incubated at 37 °C for 1 h and then centrifuged at 11 600*g* at RT for 30 min. The resulting supernatants were collected and tested for LPS content according to the manufacturer's protocol.^38^ The detected level of LPS in each NP stock solution is presented in Table S3.†

### 2.9. *In vitro* study: toxicity and cellular uptake

Human hepatic cell lines (HepG2) were cultured in DMEM medium supplemented with 10% FBS, 100 U per mL penicillin, and 100 μg per mL streptomycin. The cells were maintained in a 5% CO_2_ atmosphere at 37 °C until they reached confluency. To evaluate cell viability, HepG2 cells were incubated for 24 h with 55-GNPs/65-GNRs, both before and after conjugation with glycopeptides. The lactate dehydrogenase (LDH) assay was utilized for this assessment, as LDH is an intracellular enzyme vital to mammalian cell metabolism. Damage to the cell membrane results in the release of LDH into the extracellular environment, where it can be measured. The quantity of extracellular LDH was determined through a coupled enzymatic reaction that produces a red formazan salt, which is soluble in water. This product can be easily detected *via* UV/vis spectroscopy by measuring absorbance at 490 nm. The level of cell toxicity is directly proportional to the absorbance obtained. In all experiments, both the particle molarity and mass concentration were reported. The NP concentrations, in terms of molarity and mass, were calculated based on the UV/vis absorbance values, following the methodology outlined by Xu and colleagues.^10^

NPs' cellular uptake was evaluated by HepG2 using flow cytometry (FC) through the measurement of side scatter (SSC) values of the cell population.^39,40^ In a standard procedure, HepG2 cell lines were seeded onto 6-well plates and incubated in a cell culture incubator for 24 h at a density of 10 × 10^4^ cells per mL. Following this incubation period, the seeding medium was removed, and NPs with various coatings were immersed in both serum-free and complete culture media (the latter containing 10% FBS). These media were then added to the cell lines, which were subsequently incubated for an additional 4 hours. The mass concentration of NPs was 50 μg mL^−1^. After the treatment, cells were trypsinized, washed two times with PBS, and collected in the culture medium. To further remove the cellular debris and dead cells, the cells were also centrifuged three times at 1500*g* for 5 min and resuspended in sorting buffer (0.3 mL) composed of FBS (5%) and EDTA in PBS (1 mM). The FC measurement was performed on an Attune NxT flow cytometer equipped with a 488 nm excitation laser. Forward scatter (FSC) *vs.* side scatter (SSC) charts were recorded for both control, and particle-exposed cells. The data output of more than 20 000 events per sample was stored and analysed utilizing FlowJo software. The resulting signals were transformed into histogram plots to visualize and compare the differences in cellular uptake. The increase in SSC was calculated by subtracting the mean SSC value of the particle-treated cells from the mean SSC value of control cells. The reported values are the average of three independent measurements.

ICP-MS analysis was used to determine the amount of internalized GNPs by HepG2 cell lines.^10^ Briefly, 10 × 10^4^ HepG2 cells per mL were seeded in 6-well plates and incubated for 24 h. The cells were then treated with different coatings of 55-GNPs/65-GNRs (50 μg mL^−1^) in serum-free and FBS (10%) culture media. After that, the cells were trypsinized, washed two times with PBS, and collected in culture medium. Then, the cell samples were transferred into 1.5 mL digestion tubes, followed by the addition of HNO_3_ (0.75 mL) and HCl (0.25 mL) to each tube. The digestion process was carried out in a microwave as a sample preparation step, before quantification by ICP-MS. Before measurements, the ICP-MS setup was calibrated with a freshly prepared serial dilution of gold (Roth, Au-Standard (1000 mg mL^−1^)). The calibration curve was constructed using gold concentrations from 2 to 2500 parts per billion (ppb). The measurements were carried out with a iCAP-Q ICP-MS (Thermo Scientific, Bremen, Germany) equipped with an autosampler ASX-500 (CETAC Technologies, Omaha, USA).

### 2.10. *In vivo* study: animals and treatments

The Institute for Pharmacological Research Mario Negri IRCCS adheres to the principles set out in the following laws, regulations, and policies governing the care and use of laboratory animals: Italian Governing Law (D.lgs 26/2014; authorisation no. 19/2008-A issued March 6, 2008 by Ministry of Health); Mario Negri Institutional Regulations and Policies providing internal authorisation for people conducting animal experiments (Quality Management System Certificate-UNI EN ISO 9001:2015-Reg. No. 6121); the NIH Guide for the Care and Use of Laboratory Animals and EU directives and guidelines (EEC Council Directive 2010/63/UE). Animal studies were approved by the Mario Negri Institute Animal Care and Use Committee (IACUC) and by the Italian National Institute of Health (code no. 49/2021-PR).

Six-week-old female and male CD1 mice were obtained from Charles River (Italy) and housed under specific pathogen-free (SPF) conditions within the Institute's Animal Care Facilities. The animals were maintained in a controlled environment at a temperature of 21 ± 1 °C, a humidity of 55 ± 10%, and a 12 h light/dark cycle, with unrestricted access to food and water. A veterinarian regularly monitored the mice to ensure their well-being and to review the experimental protocols. Throughout the study, there were no reported deaths among the animals. The mice were randomly assigned to four groups, each receiving PMA-coated rods and spheres functionalized with the glycopeptide A2G2S2 (*n* = 5 per group). Each animal was administered an intravenous injection of the same GNP preparation (200 μL containing 2 × 10^11^ NPs per mouse). At designated time points (pre-injection, 1 h, 24 h, and 168 h), the mice were anesthetized, and blood samples were collected *via* retro-orbital bleeding to analyze serum toxicity markers. Following this, five mice from each group were sacrificed, and their organs were harvested for histological examination.

#### 2.10.1. Inductively coupled plasma mass spectrometry (ICP-MS)

Liver and blood samples were stored at −20 °C until digestion. Before digestion, each sample was thawed overnight and transferred to a microwave digestion vessel. Concentrated HCl (1.5 mL, 35%) and concentrated HNO_3_ (4.5 mL, 67%) were added to each vessel, maintaining a 1 : 3 ratio. The vessels were then sealed and placed in a Speedwave XPERT microwave (Berghof Products + Instruments GmbH) for digestion. Once digestion was complete, 250 μL of the resulting solution was diluted with aqua regia (2.5 mL, containing approximately 2% HNO_3_ and 0.5% HCl) and stored until analysis. The samples were analyzed using ICP-MS. Before measurement, the ICP-MS system was calibrated with a freshly prepared serial dilution of a gold standard solution (Au-Standard, 1000 mg mL^−1^, Roth). The calibration curve was created with gold concentrations ranging from 2 to 2500 parts per billion (ppb). Measurements were performed using an iCAP-ICP-MS (Thermo Scientific, Bremen, Germany) with an ASX-500 autosampler (CETAC Technologies, Omaha, USA).

#### 2.10.2. ALT/AST levels in mice serum

Hepatocellular injury and toxicity were evaluated by measuring serum alanine aminotransferase (sALT) levels. Additionally, markers of cellular toxicity, such as serum aspartate aminotransferase (sAST) activity, were assessed at 1 h, 24 h, and 168 h post-injection of NPs. sALT and sAST activities were determined using an optimized kinetic UV method according to the International Federation of Clinical Chemistry and Laboratory Medicine (IFCC) guidelines, utilizing an Aries chemical analyzer (Werfen Instrumentation Laboratory S.p.A., Italy). The results were expressed in units per liter (U L^−1^). All analyses were validated by a certified biochemical chemist and hematologist specialist using quality control sera (CQI) at the San Raffaele Mouse Clinic.

#### 2.10.3. Immunohistochemistry

At the time of autopsy, liver samples were collected from each mouse and fixed in 10% neutral buffered formalin (Bio-Optica, Italy) for at least 24 h at RT. After fixation, the samples were processed for paraffin embedding. Tissue sections, 4 μm thick, were cut using a Leica RM55 microtome (Leica Microsystem, Italy) and dried in an oven at 37 °C overnight. To visualize gold agglomerates in the liver parenchyma, autometallography (AMG) staining was performed as described in previous studies.^41,42^ Additionally, hematoxylin and eosin (H&E) staining was carried out on liver sections from both GNP-treated and vehicle-treated mice to provide an overview of tissue structure. Hematoxylin stained the cell nuclei blue, while eosin stained the extracellular matrix and cytoplasm pink. Nuclei were stained with Mayer's hematoxylin solution (Bio-Optica, Italy) for 2 min and 30 s, followed by a water rinse until color developed. Cytoplasmic staining was achieved by counterstaining with eosin Y solution (Bio-Optica, Italy) for 1 min and 20 s, followed by another rinse in tap water. The slides were then dehydrated and mounted using a xylene-based mounting medium (DPX, Sigma). IBA-1 staining was performed on liver sections from both GNP-treated and vehicle-treated mice to provide a general overview of the macrophage infiltration in tissue. IBA-1 stains macrophages brown and hematoxylin stains the cell nuclei blue. HIER was performed with citrate buffer pH = 6 for 30 min at 95 °C, followed by endogenous peroxidase inhibition with H_2_O_2_ (3%) for 10 min at RT, and incubation with a blocking solution (PBS-NGS 10%–Tween 20 0.05%) for 30 min at RT. For subcellular localization, IBA-1 (1 : 200, Wako Chemicals, USA) was used to label macrophage calcium binding protein, with the labelling system ABC kit (Vectastain Elite), and DAB (Sigma-Aldrich) used for the chromogenic reaction. Nuclei were stained with Mayer's hematoxylin solution (Bio-Optica, Italy) for 30 s, and then washed with water until the desired coloration developed. Samples were dehydrated and mounted with xylene-based mounting medium (DPX, Sigma). All images were acquired using Olympus BX61VS.

#### 2.10.4. Immunofluorescence

Immunohistochemical analysis of liver tissue was conducted using 10 μm sections cut with a cryostat. The tissue slides were fixed in 10% neutral buffered formalin (Bio-Optica, Italy) for 20 min and subsequently washed with PBS for 5 min. After washing, the sections were incubated for 1 hour in a blocking solution composed of PBS with 10% normal goat serum (NGS) and 0.1% Triton X-100, followed by another wash with PBS. For subcellular localization, the following antibodies were applied: anti-CD68 (1 : 200, Serotec, Kidlington, UK) to label lysosomes and endosomal membranes of macrophages; Lyve-1 (1 : 300, Biotechne) for labeling lysosomal endothelial cells; and CD169 (1 : 200, BioLegend) to identify the sialic acid-binding receptor 1. Additionally, Hoescht-33258 (1 μg mL^−1^ in PBS, Thermo Fisher Scientific) was used to stain the nuclei.

#### 2.10.5. Reflective confocal microscopy

To visualize the GNPs in cryo-sections of mouse liver *ex vivo*, reflection confocal microscopy was employed, as previously reported for effective gold detection. An inverted SP5 true filter-less confocal microscope from Leica Microsystems, equipped with eight laser lines and four photomultiplier tubes (PMTs), was utilized for this purpose. The 514 nm laser line was selected as the optimal excitation wavelength for gold reflection, while the reflected emission was captured through an acousto-optic tunable filter (AOTF)-driven spectral detector within the 512–517 nm wavelength range. A 1.40 NA, 63× oil immersion objective lens (Leica Microsystems) was used, allowing for optical zoom in the range of 3× to 9× when necessary. For the gold reflection scanning mode, the confocal pinhole was adjusted to 0.5 AU. During laser-scanning acquisition, both reflection scans and transmission and fluorescence biological signals were recorded, based on labeled immunofluorescence reactions. For biological signal scans, the confocal pinhole was set to 1 AU. Depending on the requirements, either a single *z*-plane acquisition at the best focal plane for each field of view or a 10-micron *z*-volume scan with a 0.25-micron *z*-step was performed. A signal resolution of 16-bit depth was achieved over a 1024 × 1024 pixel format with a 400 Hz laser acquisition frequency. Laser powers for negative control samples were set as follows: 405 nm at 10% for Hoechst signal excitation (nuclear detection), 488 nm at 25% for the CD68-(af488) signal, 514 nm at 50% for reflection modality, and 633 nm at 35% for the Lyve-1-(af647) signal. All PMTs were adjusted to a gain range of 580–690 V, with an offset range of −1/−2.5%.

For each liver section, a minimum of 15 independent fields of view were acquired to ensure proper quantification. To enhance the resolution of the reflecting signal, the Richardson–Lucy deconvolution algorithm was applied using the specific module in NIS-Elements software v5.30 (Nikon Instruments/Lim Instruments). Furthermore, for improved image quality, images were further processing through rolling-ball background suppression and median filtering algorithms (NIS-Elements v5.30). For quantification purposes, raw images without any processing were utilized. Signal thresholding, binarization, object classification, and segmentation to identify primary and secondary tissue and cell objects were performed using a custom-designed analysis pipeline within the General Analysis module in NIS-Elements v5.30.

#### 2.10.6. Transmission electron microscopy (TEM)

At the time of sacrifice, the liver was excised, reduced, and fixed in a solution of 4% paraformaldehyde and 2% glutaraldehyde in a phosphate buffer (0.12 M, pH 7.4) for 4 h at room temperature (RT). This was followed by post-fixation with 1% osmium tetroxide (OsO) in cacodylate buffer (0.12 M) for 1 h at RT. After dehydration through a graded series of ethanol, the tissue samples were cleared in propylene oxide, embedded in epoxy resin (Epon 812 Fluka), and polymerized at 60 °C for 72 h. From each embedded sample, 1 μm-thick sections were cut using a Leica EM UC6 ultramicrotome (Leica Microsystems), stained with Toluidine Blue, and mounted on glass slides for light microscopy examination. Additionally, ultra-thin sections (60 nm) from selected areas of interest were prepared, counterstained with uranyl acetate and lead citrate, and imaged using an Energy Filter Transmission Electron Microscope (EFTEM, ZEISS LIBRA® 120) equipped with a slow-scan CCD camera (Sharp eye, TRS) for enhanced imaging.

### 2.11. Statistics

All tests were conducted in triplicate or more. For *in vivo* experiments, the number of animals sacrificed at each time point was kept to a minimum (*n* = 5) in adherence to the 3Rs principle. Quantitative data are expressed as mean ± SEM. Group comparisons were performed using either an unpaired, two-tailed independent Student's *t*-test or a one-way analysis of variance (ANOVA) with repeated measures, followed by Bonferroni's post hoc test for multiple comparisons. No data were excluded from the analysis. *p*-Values are indicated in the manuscript text or shown in the figures and legends as follows: *p < 0.05, **p < 0.01, ***p < 0.001, or ****p < 0.0001. Data analysis was performed using Prism 5 software (GraphPad).

## 3. Results and discussion

### 3.1. Synthesis and characterization of glycopeptide GNPs

**Fig. 1:**
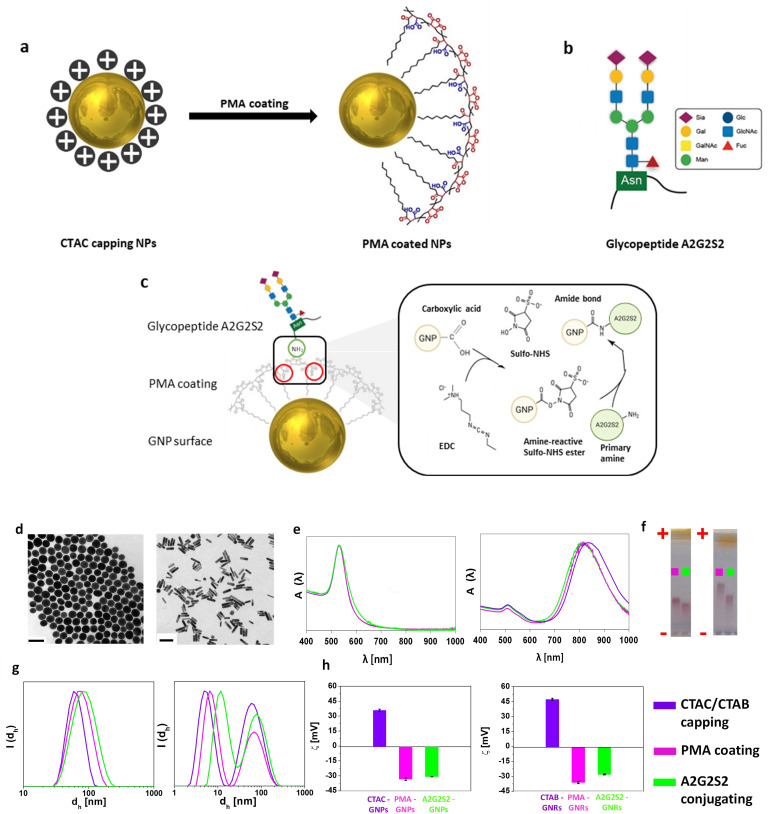
Synthesis and characterization of A2G2S2 conjugated GNPs. (a) GNPs were synthesized and stabilized by CTAC (or CTAB in the case of GNRs), which were exchanged afterwards with a negatively charged polymeric ligand (PMA) to increase the particle colloidal stability and biocompatibility. (b) Overview glycopeptide A2G2S2 is used to functionalize the GNPs with two α2-6-linked *N*-acetylneuraminic acids. (c) Schematic drawing depicting the conjugation strategy of the A2G2S2 to the PMA-coated NPs using EDC/sulfo-NHS coupling reaction. (d) Representative TEM images of 55-GNPs (left) with core diameters of *d*_c_ = 53.97 ± 4.47 nm and 65-GNRs (right) with a core length of *L*_c_ = 65.6 ± 9.4 nm and *d*_c_ = 16.01 ± 2.9 nm. Scale bars = 100 nm. Histograms of TEM images are presented in Fig. S1.† (e) Normalized absorption spectra *A*(*λ*) of 55-GNPs (left) and 65-GNRs (right). (f) Motion path of 55-GNPs before (left) and 65-GNRs (right) through agarose gel (1%) to which an electric field of 10 V cm^−1^ had been applied for 1 h. (g) Hydrodynamic diameter *d*_h_ [nm] in intensity (*d*_h_(*I*)). Three independent measurements of the intensity distribution of hydrodynamic size were presented in Fig. S2 and S3.† (h) Mean value of ζ-potential distribution *N*(*ζ*) of 55-GNPs (left) and 65-GNRs (right). The colour codes were assigned to the type of surfactant/polymer coating to the NPs, CTAC/CTAB capping (violet), PMA coating (pink), and A2G2S2 conjugating NPs (green).

CTAC/CTAB capped 55-GNPs/65-GNRs were synthesized using well-established protocols (Fig. 1a).^31,32^ To remove the toxic surfactants (CTAC or CTAB), the NPs were subjected to a round-trip phase transfer from water to chloroform using both DDA and PEG 2k, and then, back to water using polymer coating techniques. The PMA-coated 55-GNPs/65-GNRs were conjugated with the glycopeptide A2G2S2 through EDC/sulfo-NHS chemistry (Fig. 1b and c). The resulting GNPs had a core diameter (*d*_c_) of 53.9 ± 4.4 nm for spherical NPs, while for the gold nanorods, the dimensions were *d*_c_ = 16.01 ± 2.9 nm and core length (*L*_c_) = 65.6 ± 9.4 nm as confirmed by TEM (Fig. 1d). Approximately 55 nm GNPs and *ca.* 65 nm GNRs were selected based on reproducible synthesis protocols in the literature,^10,31,32^ and their known shape-dependent biodistribution.^10,43,44^ Prior studies have shown that nanoparticle curvature affects ligand density and binding affinity,^43,45^ and our earlier work supports the use of these dimensions to evaluate glycopeptide-mediated effects.^42^ As shown in Fig. 1e, no significant change in the position or the shape of the plasmon band (SPR) was observed after conjugation with the A2G2S2 glycopeptide. However, the successful coupling of A2G2S2 to PMA-coated NPs was confirmed by gel electrophoresis as shown in Fig. 1f; the A2G2S2 attachment involved the formation of an amide bond between the free primary amine present in the glycopeptide and the free carboxylic acids in the PMA-coated NPs, delaying the NP migration due to the increase in their hydrodynamic size (Fig. 1g), and decrease in their negative charge (Fig. 1h), which was further confirmed by DLS and LDA, respectively.

### 3.2. Evaluation of the targeting specificity of the glycopeptides

To study the targeting properties of A2G2S2 glycopeptides, the Sambucus Nigra lectin (SNA), a protein with a high binding affinity to sialic acid terminal end bound *via* α-2,6 linkage, was used to evaluate whether the glycan conjugates on the NP surface, were accessible for binding. The A2G2S2 conjugated PMA_55-GNPs/65-GNRs were dispersed in PBS in the presence of SNA (100 μg mL^−1^). As a negative control, the same amount of – PMA_55-GNPs/65-NRs was incubated in the presence of SNA using the same conditions. As shown in Fig. S4(a and b),† after the incubation of PMA_55-GNPs/65-GNRs with SNA, no changes were observed in the SPR peaks, suggesting no selective binding. However, the addition of SNA to A2G2S2_55-GNPs/65-GNRs led to selective aggregation of the GNPs. This was confirmed by a broadening and a redshift (∼5 nm) of the SPR peak and a decrease in the overall absorbance (Fig. S4c and d,† left side). These results agreed with the DLS measurement (Fig. S4c and d,† right side), where an increase in the hydrodynamic size of A2G2S2 conjugated PMA_55-GNPs/65-GNRs after incubation with SNA confirmed the selective targeting.

**Fig. 2:**
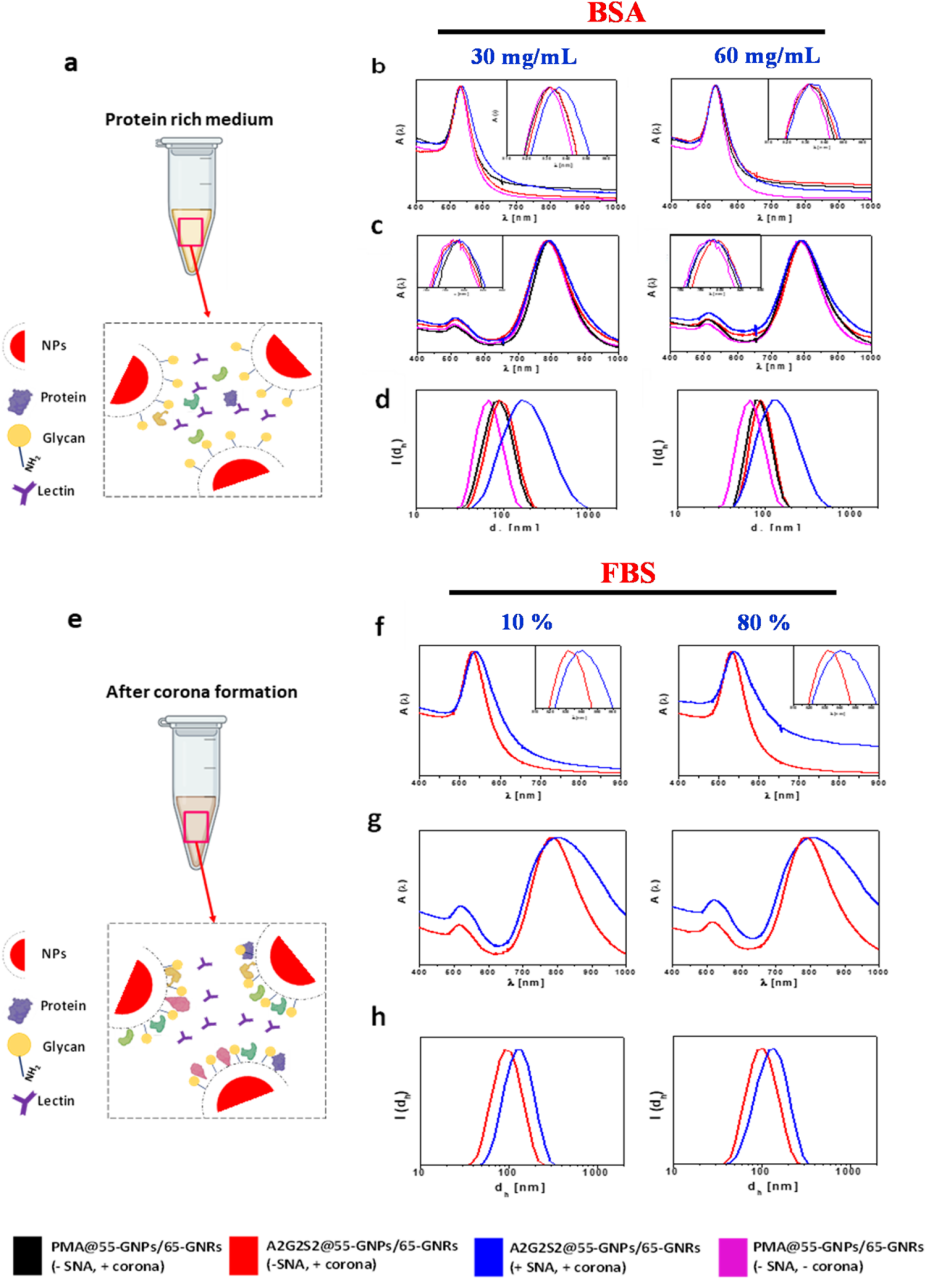
Targeting properties of A2G2S2-conjugated gold nanomaterials. (a) Overview of competitive binding between BSA and SNA. (b and c) Normalized UV-vis absorbance spectra of (b) 55-GNPs and (c) 65-GNRs after incubation in different concentrations of bovine serum albumin (BSA), 30 mg mL^−1^ (left) and 60 mg mL^−1^ (right), in the absence and presence of SNA. (d) Hydrodynamic diameter *d*_h_ [nm] by intensity (*d*_h_(*I*)) of 55-GNPs after incubation with different concentrations of bovine serum albumin (BSA), 30 mg mL^−1^ (left) and 60 mg mL^−1^ (right), in the absence and presence of SNA. (e) Scheme describing the lectin binding assay after hard corona formation from FBS. (f and g) Normalized UV-vis absorbance spectra of 55-GNPs (f) and 65-GNRs (g) after incubation with different concentrations of FBS 10% (left) and 80% (right) and further incubated at 37 °C for 1 h in the absence and presence of SNA. (h) Hydrodynamic diameter *d*_h_ [nm] in intensity (*d*_h_(*I*)) of the same 55-GNPs presented in (f) FBS 10% (left) and 80% (right). Colour codes are assigned to PMA-coated NPs (pink: control sample, no SNA, no corona, water dispersible), PMA_55-G NPs/65-GNRs (black: *in situ* corona – no SNA), A2G2S2_55-GNPs/65-GNRs (red: *in situ* corona – no SNA), and A2G2S2_55-GNPs/65-GNRs (blue: *in situ* corona – with SNA). Insets show normalised spectra for the SPR shift.

To investigate the retention of the targeting properties of A2G2S2 glycans in protein-rich environments, A2G2S2-conjugated PMA_55-GNPs/65-GNRs were subjected to incubation with varying concentrations of BSA at 30 and 60 mg mL^−1^, both in the presence and absence of SNA, over 24 hours (Fig. 2a). As a negative control, non-glycosylated PMA_55-GNPs/65-GNRs were similarly incubated with BSA (without SNA). As depicted in Fig. 2(b–d), the interaction of biomolecules (forming a protein corona) with the NP surface resulted in a slight shift in the position of the SPR band (inset, Fig. 2b and c) and an increase in the hydrodynamic size (Fig. 2d) of PMA_55-GNPs/65-GNRs or their A2G2S2-conjugated counterparts upon exposure to BSA (without SNA). Notably, A2G2S2-conjugated PMA_55-GNPs/65-GNRs exhibited a more pronounced red shift and a significant increase in hydrodynamic size following exposure to BSA (with SNA), indicating the biological accessibility of A2G2S2 in protein-rich media. Interestingly, the shape of the SPR peak of A2G2S2-conjugated NPs showed minimal change after incubation with BSA (with SNA), suggesting the absence of aggregation. This observation was further supported by DLS analysis and SDS-PAGE, where the binding of SNA to the particle surface was detectable in the gel (Fig. S5†).

We then evaluated whether the specificity of A2G2S2-conjugated NPs towards lectin binding remained preserved in a more complex protein milieu, such as FBS (10% and 80%) and human blood plasma (hBs), incubated for 1 h at 37 °C. The GNPs were then centrifuged and carefully washed 3 times with PBS to remove weakly attached and unbound biomolecules. After that, the adsorbed proteins were eluted from the surface of the NPs and characterized by SDS-PAGE. As shown in Fig. S6,† the eluted bound proteins from all NPs showed no significant differences in banding patterns, displaying bands in the range of 52–95 kDa.

To further explore the effect of protein corona on the targeting ability of A2G2S2 conjugated PMA_55-GNPs/65-GNRs, we incubated them in different percentages of FBS (10% and 80%) for 1 h at 37 °C. After that, the GNPs were subjected to an extensive and careful washing process using PBS to remove unbound and weakly attached proteins. This treatment leaves a layer of proteins (known as the hard corona, HC) that have high affinity and are strongly attached to the particle surface. The resulting HC–NP complexes were then dispersed in PBS, followed by the addition of 10 μg of SNA and further incubated for 1 h at 37 °C without shaking. A positive control of A2G2S2 conjugated PMA_55-GNPs/65-GNRs was incubated in FBS (10% and 80%) in the absence of SNA (Fig. 2e). As shown in Fig. 2f–h, a redshift in the SPR of A2G2S2 conjugated PMA_55-GNPs/65-GNRs and an increase in the hydrodynamic size of A2G2S2 conjugated PMA_55-GNPs were detected after the treatment with SNA. These data suggest that the glycans are biologically accessible and capable of binding receptors even after the biomolecular corona formation, which is an important feature that can be exploited in the field of nanomedicine.

### 3.3. *In vitro* cytotoxicity and cellular uptake study

**Fig. 3:**
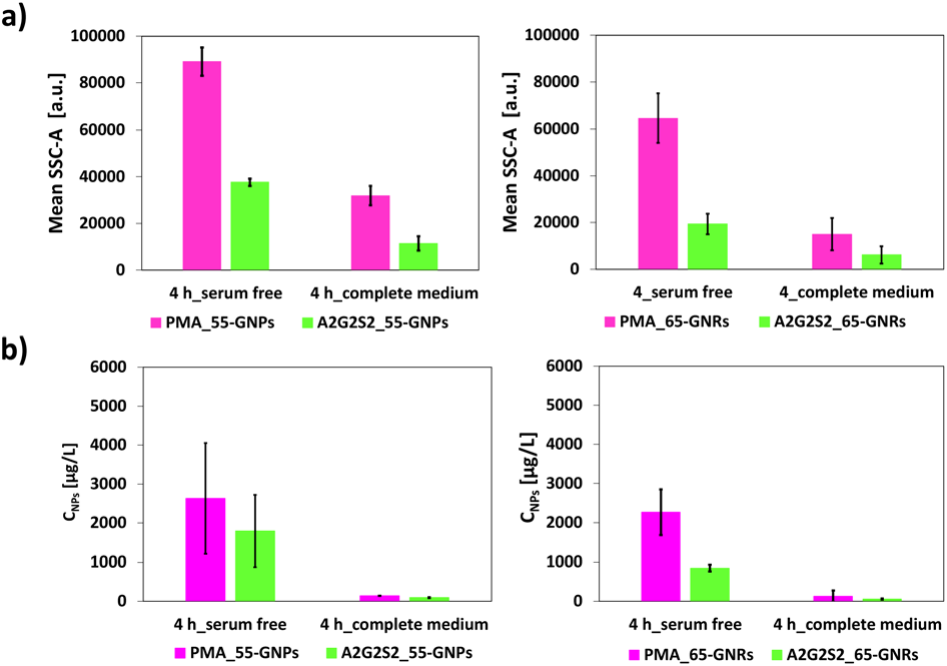
GNP cell uptake in the HepG2 cell line. (a) The mean of the flow cytometry side scattering intensity (SSC-A) of HepG2 cell lines treated with PMA_55-GNPs/65-GNRs (pink colour) and their conjugates with A2G2S2 (green colour) in serum-free (left) and 10% FBS (right) culture media for 4 h. The data were calculated from the statistical analysis in Fig. S7.† (b) Amount of elemental Au found in HepG2 after exposure to PMA_55-GNPs/65-GNRs (pink colour) and their conjugates with A2G2S2 (green colour) in serum-free (left) and 10% FBS (right) culture media for 4 h. The amount of Au was determined using ICP-MS.

An *in vitro* assessment was conducted to evaluate the toxicity of PMA_55-GNPs/65-GNRs and their conjugates with A2G2S2, as well as to investigate whether the presence of glycans affected cellular uptake. To achieve this, we used the HepG2 cell line due to its similarity to primary hepatocytes.^46–48^ HepG2 cells were exposed to a broad concentration range of GNPs (before and after A2G2S2 conjugation) for 24 h by using the limiting dilution analysis (LDA). No effect on cell viability (Fig. S7†) or sign of acute toxicity was observed even at the higher concentration levels (200 μg per mL NPs) for all types of NPs used. However, a difference in the uptake profile was observed by flow cytometry and ICP-MS when cells were exposed to glycan-conjugated and PMA-coated NPs at two different time points. Interestingly, the presence of glycans affected the cellular uptake rate; a lower cellular uptake was observed in the glycosylated NPs with A2G2S2 compared to the PMA-coated NPs (non-glycosylated) (Fig. 3 and S8†), with stronger uptake reduction observed in the glycosylated GNRs. It is worth mentioning that the cellular uptake of NPs was lower in the 10% FBS-supplemented media compared to the serum-free media, which can be attributed to protein corona formation.^49,50^

### 3.4. Influence of A2G2S2 conjugation on hepatic biodistribution and toxicity of GNPs

Then, an *in vivo* study to evaluate whether the glycans decorating the NP surface could have an effect on their biodistribution was carried out. While the physico-chemical studies confirmed successful glycan conjugation on both surfaces, *in vitro* studies showed that the GNRs' cellular uptake was predominantly affected. In this study, we focused on the gold nanorods due to their small curvature, which would facilitate their interaction in the biological system.^51–53^ For this experiment, a total concentration of 2 × 10^11^ NPs was injected intravenously into each mouse in a single dose. This dosage was selected because it aligns with those used in previous studies^42^ and did not result in any observed toxicity. Samples were collected and analyzed at three different time points: 1 hour, 1 day, and 7 days post-injection. This was done to assess potential toxicity and to quantify the presence of GNPs in both circulation and the liver.

**Fig. 4:**
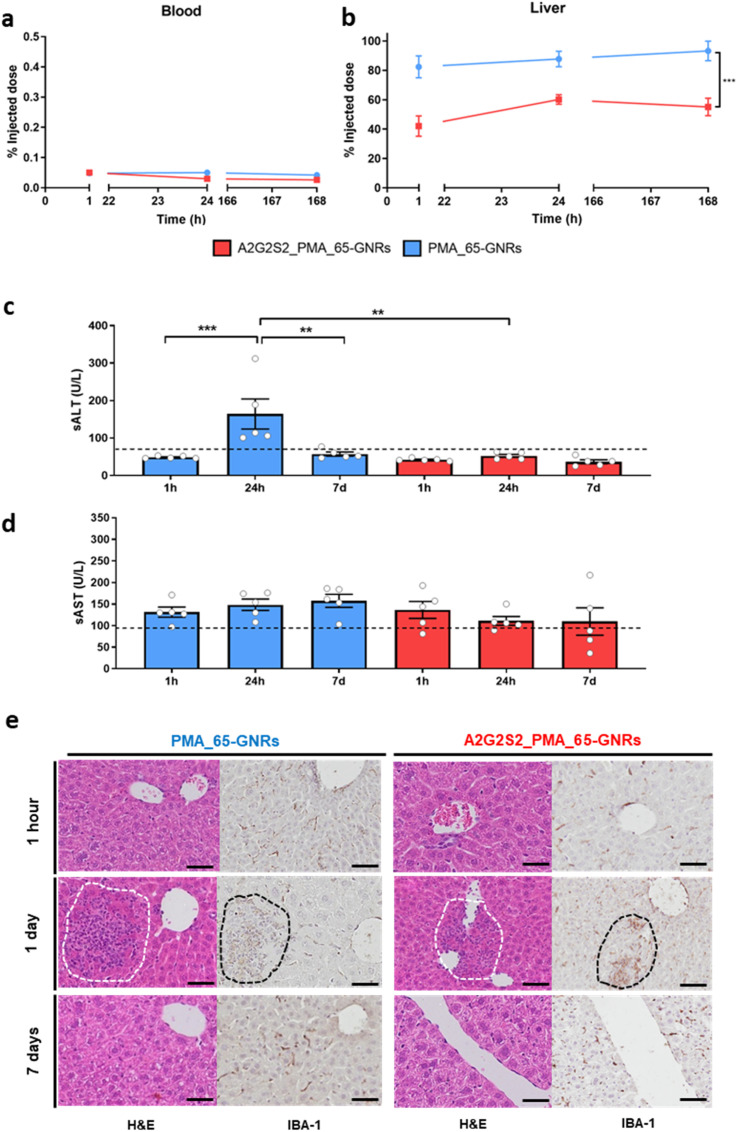
Biodistribution and toxicity of a single dose administration of glyco-GNRs coated with glycopeptide A_2_G_2_S_2_ in healthy mice. (a and b) Percentage of the injected dose (% ID) measured by ICP-MS in the blood (a) and in liver (b) derived from mice treated with 4 different GNPs at 1, 24 and 168 (7 days) hours after treatment. The data are reported as mean ± s.e.m. One-way ANOVA followed by the Bonferroni post hoc test was carried out. Significant difference (**p < 0.01 and ***p < 0.001) comparing the different shapes and glycopeptide functionalization at the same time point. (c) Hepatic transaminase levels in serum. Activity (units per liter [U L^−1^]) of (c) sALT and (d) sAST measured at the indicated time points in groups of mice injected with PMA-55-GNPs/65-GNRs compared to the A2G2S2 conjugated NPs. Dashed lines indicate the upper value of normality of each variable (70 U L^−1^ for sALT and 83 U L^−1^ for sAST). Data are presented as mean ± s.e.m. *P* values were determined by one-way ANOVA with Bonferroni's correction (***p < 0.001 and ***p < 0.001). (e) Liver histology by H&E and IBA-1 staining in livers treated with GNPs after 1 hour, 1 and 7 days. Dashed lines indicate the macrophage infiltration in the liver parenchyma. Scale bars = 50 μm.

Despite the incorporation of Sia, the A2G2S2 modification did not extend the circulation lifetime of the GNPs. They were rapidly eliminated from the bloodstream, with clearance occurring in less than an hour (Fig. 4a). Additionally, the presence of Sia did not prevent the opsonization of proteins on the NP surface. However, a difference in liver accumulation was observed with the glycosylated NPs in the liver. PMA_65-GNRs accumulated inside the liver in less than 1 hour (Fig. 4b). Rod-like NPs undergo faster clearance from blood, with non-glycosylated 65-GNR levels oscillating from 80% ID after 1 hour and increasing nearly to 98% ID after 7 days (Fig. 4b). In contrast, A2G2S2 conjugated 65-GNRs showed lower liver retention, with only 50% ID detected after 7 days of injection.

To evaluate whether A2G2S2 may induce liver toxicity, serological levels of alanine transaminase (sALT) and aspartate transaminase (sAST), specific markers for liver injury or cell death, respectively, were quantified to detect possible necroinflammation. As is shown in Fig. 4c, glycosylated NPs did not alter the enzymatic values as all GNRs were found below the maximum level of normality for sALT, 70 U L^−1^ (dashed line), or sAST (Fig. 4d), 83 U L^−1^ (dashed line). However, a significant increase after 24 hours was observed for non-glycosylated 65-GNRs, overpassing the maximum value of normality for ALT (70 U L^−1^) (Fig. 4c).

Histological evaluation with H&E and IBA-1 staining of liver tissues after 1 hour, 1, and 7 days (Fig. 4e) revealed acute inflammation after 24 h, with lymphocyte infiltration near the portal triad (PT) that was not observed in CTRL mice (Fig. S9†). IBA-1 staining confirmed the presence of macrophages in the inflamed foci, showing the implication of macrophages in the initial steps of hepatic inflammation due to the recruitment of liver residential macrophages (Kupffer cells) and monocytes from the circulation (Fig. 4e). Interestingly, the inflammation disappeared after 7 days, demonstrating that it was a transitory event and suggesting the absence of liver dysfunction. No macroscopic changes in organs, weight loss, diarrhea, inability to walk, or other types of clinical signs were observed in mice, demonstrating the transitory inflammatory response.

**Fig. 5:**
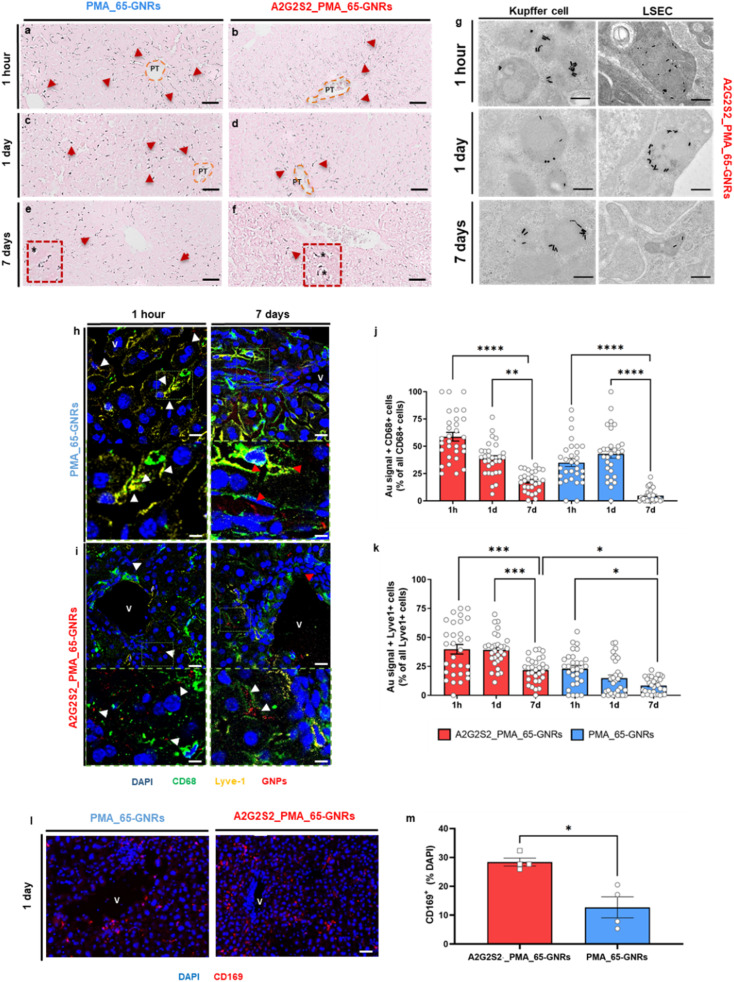
Histological evaluation of gold distribution (black spots) in liver tissue by AMG. (a and f) Representative micrographs of livers from mice treated with A2G2S2_65-GNRs (a, c and e) and A2G2S2_65-GNRs (b, d and f) and sacrificed after (a and b) 1 hour, (c and d) 1 day and (e and f) 7 days after NP administration. Red arrows show silver-stained NPs inside hepatic cells. Orange dotted lines show NPs circulating from the portal triad (PT). Black asterisks show silver-stained NPs magnified inside hepatic cells. Scale bars for AMG images = 100 μm (large image) and 20 μm (small insets). (g) Representative TEM images of A2G2S2_65-GNRs in healthy mice treated at three different time points (1 hour, 1 day and 7 days). Images showed GNPs internalized in lysosomes (small insets) of Kupffer cells and LSECs. Scale bars for TEM images = 1 μm (large image) and 200 nm (small insets). (h and i) Reflective SIM in liver sections from healthy mice treated with glyco-NPs, (h) PMA_65-GNRs and (i) A2G2S2_PMA_65-GNRs at two different time points (1 hour and 7 days). In blue, the nuclei (Hoescht) are shown, in green the macrophages (CD68) are shown, in yellow, the lysosomal endothelial cells (Lyve-1) are shown and in red, GNPs (Au-reflective signal) are shown. White arrowheads show reflected Au signals in the inter-cellular space near macrophages (CD68^+^ cells) and red arrowheads indicate intra-cellular localization. (V) Vessel with the Au-signal. Scale bars = 25 μm (large images), 5 μm (small insets). (j and k) Quantification of the area occupied by GNPs in (j) CD68^+^ and (k) Lyve-1^+^ cells in liver sections from treated healthy mice at three different time points (1 hour, 7 days and 47 days). Data are presented as mean ± s.e.m. *P* values were determined by one-way ANOVA with Bonferroni's correction *p < 0.05, **p < 0.01, ***p < 0.001 and ****p < 0.0001. (l) Immunostaining in liver sections from healthy mice treated with PMA_65-GNRs with and without the glycopeptide after 1 day. In blue, the nuclei (Hoescht) are shown and in red, CD169 (Siglec-1) is shown. (V) Vessel. Scale bars = 25 μm. (m) Quantification of the area occupied by CD169 in liver sections from treated healthy mice after 1 day. Data are presented as mean ± s.e.m. *P* values were determined by one-way ANOVA with Bonferroni's correction *p < 0.05.

Qualitative histological studies through autometallography silver staining (AMG) (Fig. 5) confirmed the presence of nanomaterials in the liver at all time points, supporting the ICP-MS findings and revealing the biodistribution of GNPs inside hepatic cells. Polarized and elongated spots were visible in the liver of mice sacrificed 1 hour after the treatment (red arrows), with most signals located near the portal triad areas (dotted lines), due to early interaction of the GNPs with LSECs as they enter from the circulation (Fig. 5a and b). Interestingly, A2G2S2_65-GNRs were localized near the PT during the first hour and then, the GNR signal extended throughout the liver parenchyma over time (Fig. 5a–e). However, the A2G2S2_65-GNRs signal was predominantly localized in the surrounding areas of the PT at all-time points, with some GNRs in the free parenchymal areas (Fig. 5b–f). This is because A2G2S2_65-GNRs were not distributed all over the hepatic tissue, due to a fast interaction with hepatic cells, such as KCs or LSECs present in the walls of the sinusoids that bloodstream, resulting in very clear accumulation near the PT. Representative TEM images confirmed the GNPs exclusively inside KCs and LSECs with no sign of NPs in the hepatocytes or liver parenchyma (Fig. 5g). Indeed, all A2G2S2_65-GNRs were well preserved and mostly found as clusters inside secondary lysosomes. The internalization did not affect the ultrastructural bloodstream, causing a very clear accumulation near the PT. Representative TEM images confirmed the exclusive localization of GNPs inside KCs and LSECs with no sign of NPs in the hepatocytes or the liver parenchyma (Fig. 5g). Indeed, all A2G2S2_65-GNRs were well preserved and mostly found as clusters inside secondary lysosomes. The internalization did not affect the ultrastructural organization of both KCs and LSECs, showing perfectly preserved organelles such as the endoplasmic reticulum, Golgi apparatus, nucleus, and numerous primary lysosomes.

To obtain quantitative data related to GNRs' co-localization with KCs and LSECs, structured illumination microscopy (SIM) was used to detect the reflected light from the GNP surface by using fluorescent antibodies against CD68 and Lyve-1, respectively (Fig. 5h–k). Clusters of GNRs were observed at all-time points in the peripherical areas around CD68^+^ cells and Lyve1^+^ cells (Fig. 5h and i). Interestingly, the signal from PMA_65-GNRs was distributed throughout the parenchyma and internalized by hepatic cells (Fig. 5h); meanwhile, A2G2S2_PMA_65-GNRs functionalized with the glycopeptide were predominantly localized near the vessels (Fig. 5i), as was also observed by AMG. During the first hour, aggregates of both GNRs were found in the intercellular space surrounding mostly CD68^+^ cells (white arrowheads). However, after 7 days, aggregates of PMA_65-GNR reflective structures were found in the lysosomes, forming little clusters of laser-reflecting structures within CD68^+^ cells and Lyve1^+^ cells (red arrowheads). Meanwhile, A2G2S2_PMA_65-GNR reflective structures were visible in the intercellular spaces surrounding CD68^+^ cells and Lyve1^+^ cells (white arrowheads). The total percentage of Lyve-1 and CD68 cells reflecting from internalized glycosylated-GNRs was very similar between both NPs (Fig. 5j and k). PMA_65-GNRs formed clusters inside hepatic cells due to internalization by endocytosis; meanwhile the A2G2S2_PMA_65-GNRs made small clusters around phagocytic cells, maybe due to the presence of the glycopeptide on the GNP surface, resulting in slower uptake by phagocytosis.^54^

To probe the specificity of Sia for Siglecs expressed in immune cells such as macrophages, the CD169 (Siglec-1) expression in hepatic tissues was quantified with immunostaining. As expected, the presence of the glycopeptide caused a significant increase in the expression of CD169 in hepatic cells compared with PMA_65-GNRs (Fig. 5l and m). Here, we have shown that the GNP surface display of Sia-containing glycopeptides facilitated reduced liver uptake. This is because Sia acts as a mask, hiding recognition sites from the glycan chains, such as galactose, other proteins, or macromolecules, which helps evade immune cells and modifies the protein corona composition. However, Sia itself is also a biological recognition site, being identified by a large variety of molecules such as lectins, antibodies, or hormones.^55^ Specifically, cells from the immune system present a high variety of Siglecs, depending on the cell population, KCs express the receptor sialoadhesin (Siglec-1) on its surface.^56,57^ Meanwhile, LSECs have been shown to express Siglec-E,^34^ confirming the specific targeting properties of glycosylated GNRs. Our data suggest that KCs and LSECs are the predominant targeted cell populations by α-2,3 linked Sia-GNRs. Lastly, an increase in α2–6 sialylation has been strongly implicated in cancer metastasis, as this modification promotes reduced recognition by the immunological cell and enhanced circulation.

While this study focuses on the impact of glycopeptide functionalization and nanoparticle geometry on liver biodistribution and immune cell interactions, further research is needed to fully understand the fate of these nanoparticles. Future studies should investigate clearance routes such as renal and hepatobiliary pathways, and the role of the mononuclear phagocyte system. In addition, *ex vivo* imaging of organs and urine analysis could provide complementary insights. Computational modelling may also help clarify how glycopeptides interact with plasma proteins and how nanoparticle shape affects corona formation and ligand exposure.

## 4. Conclusions

Nanomaterial clearance from the bloodstream and unspecific sequestration inside the liver is a known barrier to the clinical translation of nanomedicine. Herein, we developed a strategy to overcome the liver barrier by mimicking nature, using a natural glycopeptide predominantly expressed in human cells that presents terminal Sias in its structure. While previous studies have mainly focused on terminal sialylation using monosaccharide units, the use of the glycosylated peptide enables the correct glycan orientation on the GNP surface, and the use of α2–6 linkage ensures high specificity towards sialic acid receptors and reduces immunological recognition. Sia expression reduced GNR uptake by up to 40%, due to a higher exposure of ligands on the flat surface, which protected GNPs from opsonisation by specific proteins that may help immune cells remove the nanomaterials. *In vivo*, the nanomaterials were predominantly observed in KCs and LSECs, indicating that the presence of Sia enhances the masking effect. This study is a detailed proof-of-concept that functionalization with Sia-expressing glycans may be key for enabling nanomaterials to escape the “hepatic blackbox”, providing a foundation for other studies to improve the targeting efficiency of nanomaterials in diseased tissues by evading RES organs.

## Author contributions

Mahmoud G. Soliman: conceptualization, investigation, funding acquisition, validation, visualization, writing – original draft, writing – review and editing. Jennifer Fernandez Alarcon: investigation, validation, writing – original draft, writing – review and editing. Tanja Ursula Lüdtke, Martina B. Violatto, Marko Dobricic, Chiara Cordiglieri, Alessandro Corbelli, Fabio Fiordaliso and Giovanni Sitia: investigation, writing – review and editing. James S. O'Donnell, Daniel IR Spencer and Sergio Moya: Writing – review and editing. Paolo Bigini and Marco P Monopoli: conceptualization, funding acquisition, resources, supervision, writing – review and editing.

## Conflicts of interest

Daniel IR Spencer is employed by Ludger Ltd, a company that specializes in the commercialization of glycan analytics and standards. The other authors declare no conflicts of interest.

## Supplementary Material

NA-OLF-D5NA00464K-s001

## Data Availability

The data supporting this article have been included as part of the ESI.† Raw data are also accessible from the corresponding author upon reasonable request.

## References

[cit1] Ali M. R. K., A Rahman M., Wu Y., Han T., Peng X., Mackey M. A., Wang D., Shin H. J., Chen Z. G., Xiao H. (2017). Proc. Natl. Acad. Sci. U. S. A..

[cit2] von Maltzahn G., Park J.-H., Agrawal A., Bandaru N. K., Das S. K., Sailor M. J., Bhatia S. N. (2009). Cancer Res..

[cit3] Cheng Y., C Samia A., Meyers J. D., Panagopoulos I., Fei B., Burda C. (2008). J. Am. Chem. Soc..

[cit4] Kotcherlakota R., Nimushakavi S., Roy A., Yadavalli H. C., Mukherjee S., Haque S., Patra C. R. (2019). ACS Biomater. Sci. Eng..

[cit5] Bailly A. L., Correard F., Popov A., Tselikov G., Chaspoul F., Appay R., Al-Kattan A., Kabashin A. V., Braguer D., Esteve M. A. (2019). Sci. Rep..

[cit6] Haute D. V., M Berlin J. (2017). Ther. Deliv..

[cit7] Jong W. H. D., Hagens W. I., Krystek P., C Burger M., Sips A. J. A. M., Geertsma R. E. (2008). Biomaterials.

[cit8] Zhang Y. N., Poon W., Tavares A. J., McGilvray I. D., Chan W. C. W. (2016). J. Controlled Release.

[cit9] Singer A. L., Adlersberg L., Sadek M. (1972). J. Reticuloendothel. Soc..

[cit10] Xu M., Soliman M. G., Sun X., Pelaz B., Feliu N., Parak W. J., Liu S. (2018). ACS Nano.

[cit11] Boselli L., Castagnola V., Armirotti A., Benfenati F., Pompa P. P. (2024). Small.

[cit12] Salvati A., Pitek A. S., Monopoli M. P., Prapainop K., Bombelli F. B., Hristov D. R., Kelly P. M., Åberg C., Mahon E., Dawson K. A. (2013). Nat. Nanotechnol..

[cit13] Dai Q., Yan Y., Guo J., Björnmalm M., Cui J., Sun H., Caruso F. (2015). ACS Macro Lett..

[cit14] Perry J. L., Reuter K. G., Kai M. P., Herlihy K. P., Jones S. W., Luft J. C., Napier M., Bear J. E., DeSimone J. M. (2012). Nano Lett..

[cit15] Zhang G., Yang Z., Lu W., Zhang R., Huang Q., Tian M., Li L., Liang D., Li C. (2009). Biomaterials.

[cit16] Shi L., Zhang J., Zhao M., Tang S., Cheng X., Zhang W., Li W., Liu X., Peng H., Wang Q. (2021). Nanoscale.

[cit17] Niidome T., Yamagata M., Okamoto Y., Akiyama Y., Takahishi H., Kawano T., Katayama Y., Niidome Y. (2006). J. Contr. Release.

[cit18] Knop K., Hoogenboom R., Fischer D., Schubert U. S. (2010). Angew. Chem., Int. Ed..

[cit19] Hong R. L., Huang C. J., Tseng Y. L., Pang V. F., Chen S. T., Liu J. J. (1999). Clin. Cancer Res..

[cit20] Sun C.-Y., Shen S., Xu C.-F., Li H.-J., Liu Y., Cao Z.-T., Yang X.-Z., Xia J.-X., Wang J. (2015). J. Am. Chem. Soc..

[cit21] García I., Iglesias A. S., Lacey M. H., Grzelczak M., Penadés S., Liz-Marzán L. M. (2015). J. Am. Chem. Soc..

[cit22] Kim Y.-H., Min K. H., Wang Z., Kim J., Jacobson O., Huang P., Zhu G., Liu Y., Yung B., Niu G., Chen X. (2017). Theranostics.

[cit23] Compostella F., Pitirollo O., Silvestri A., Polito L. (2017). Beilstein J. Org. Chem..

[cit24] Ohtsubo K., Marth J. D. (2006). Cell.

[cit25] Clerc F., Reiding K. R., Jansen B. C., Kammeijer G. S. M., Bondt A., Wuhrer M. (2016). Glycoconj. J..

[cit26] Varki A. (2008). Trends Mol. Med..

[cit27] Schauer R., Kelm S. (1997). Int. Rev. Cytol..

[cit28] Xia Y., Zhong J., Zhao M., Tang Y., Han N., Hua L., Xu T., Wang C., Zhu B. (2019). Drug Deliv..

[cit29] Chen W. C., Sigal D. S., Saven A., Paulson J. C. (2011). Leuk. Lymphoma.

[cit30] Park E. I., Manzella S. M., Baenziger J. U. (2003). J. Biol. Chem..

[cit31] Hanske C., Rubio G. G., Hamon C., Formentín P., Modin E., Chuvilin A., Martínez A. G., Marsal L. F., Liz-Marzán L. M. (2017). J. Phys. Chem. C.

[cit32] Ye X., Caglayan H., Chen J., Xing G., Zheng C., Nguyen V. D., Kang Y., Engheta N., Kagan C. R., Murray C. B. (2012). ACS Nano.

[cit33] Soliman M. G., Pelaz B., Parak W. J., del Pino P. (2015). Chem. Mater..

[cit34] Lin C.-A. J., Sperling R. A., Li J. K., Yang T.-Y., Li P.-Y., Zanella M., Chang W. H., Parak W. J. (2008). Small.

[cit35] Soliman M. G., Davies H. A., Sharkey J., Levy R., Madine J. (2022). PLoS One.

[cit36] Xu M., Soliman M. G., Sun X., Pelaz B., Feliu N., Parak W. J., Liu S. (2018). ACS Nano.

[cit37] Haiss W., Thanh N. T. K., Aveyard J., Fernig D. G. (2007). Anal. Chem..

[cit38] Konwar A., Chowdhury D., Dan A. (2019). Mater. Chem. Front..

[cit39] Wu Y., Ali M. R. K., Dansby K., El-Sayed M. A. (2019). Anal. Chem..

[cit40] Sun X., Soliman M. G., Nold P., Said A., Chakraborty I., Pelaz B., Schmied F., von Pückler K., Figiel J., Zhao Y., Brendel C., Hassan M., Parak W. J., Feliu N. Appl. Mater. Today.

[cit41] Talamini L., Violatto M. B., Cai Q., Monopoli M. P., Kantner K., Krpetić Z., Potti A. P., Cookman J., Garry D., Silveira C. P., Boselli L., Pelaz B., Serchi T., Cambier S., Gutleb A. C., Feliu N., Yan Y., Salmona M., Parak W. J., Dawson K. A., Bigini P. (2017). ACS Nano.

[cit42] Alarcon J. F., Soliman M. G., Lüdtke T. U., Clemente E., Dobricic M., Violatto M. B., Corbelli A., Fiordaliso F., Cordiglieri C., Talamini L., Sitia G., Moya S., Bigini P., Monopoli M. P. (2023). Nanoscale.

[cit43] Devika Chithrani A. A. G. B., Chan W. C. W. (2006). Nano Lett..

[cit44] Dykmana L., Khlebtsov N. (2012). Chem. Soc. Rev..

[cit45] Mei B. C., Oh E., Susumu K., Farrell D., Mountziaris T. J., Mattoussi H. (2009). Langmuir.

[cit46] Kawata K., Osawa M., Okabe S. (2009). Environ. Sci. Technol..

[cit47] Arzumanian V. A., Kiseleva O. I., Poverennaya E. V. (2021). Int. J. Mol. Sci..

[cit48] Tkachenko A. G., Xie H., Coleman D., Glomm W., Ryan J., Anderson M. F., Franzen S., Feldheim D. L. (2003). J. Am. Chem. Soc..

[cit49] Lee Y. K., Choi E.-J., Webster T. J., Kim S.-H., Khang D. (2015). Int. J. Nanomed..

[cit50] Yan Y., Gause K. T., Kamphuis M. M. J., Ang C.-S., O'Brien-Simpson N. M., Lenzo J. C., Reynolds E. C., Nice E. C., Caruso F. (2013). ACS Nano.

[cit51] Solveyra E. G., Szleifer I. (2015). Wiley Interdiscip. Rev. Nanomed. Nanobiotechnol..

[cit52] Toy R., Peiris P. M., Ghaghada K. B., Karathanasis E. (2014). Nanomedicine.

[cit53] Cooley M., Sarode A., Hoore M., Fedosov D. A., Mitragotrib S., Gupta A. S. (2018). Nanoscale.

[cit54] Behzadi S., Serpooshan V., Tao W., Hamaly M. A., Alkawareek M. Y., Dreaden E. C., Brown D., M Alkilany A., C Farokhzad O., Mahmoudi M. (2017). Chem. Soc. Rev..

[cit55] Schauer R. (2009). Curr. Opin. Struct. Biol..

[cit56] Crocker P. R., Paulson J. C., Varki A. (2007). Nat. Rev. Immunol..

[cit57] Macauley M. S., Crocker P. R., Paulson J. C. (2014). Nat. Rev. Immunol..

