# Supplementary material for: Decoration of gold nanoparticles with glycopeptides leads to a lower cellular uptake and liver retention
Source: Nanoscale Adv. 2025 Aug 12;7(18):5784–98. doi: 10.1039/d5na00464k (PMC12341466; doi:10.1039/d5na00464k)
Supplement: NA-007-D5NA00464K-s001 [file NA-007-D5NA00464K-s001.pdf]

Supplementary Information (SI) for Nanoscale Advances.
This journal is © The Royal Society of Chemistry 2025

Decoration of gold nanoparticles with glycopeptides leads to a lower cellular uptake and liver retention

Mahmoud G. Soliman[1,2‡], Jennifer Fernandez Alarcon[3‡], Tanja Ursula Lüdtke[4], Martina B. Violatto[3], Marko Dobricic[1], Chiara Cordiglieri[5], Alessandro Corbelli[3], Fabio Fiordaliso[3], Giovanni Sitia[6], James S. O'Donnell[7], Daniel IR Spencer[8],Sergio Moya[4], Paolo Bigini[3], and Marco P Monopoli[1*]*

[1]Department of Chemistry, Royal College of Surgeons of Ireland RCSI, St Stephens Green 123, Dublin, Ireland.

[2]Physics Department, Faculty of Science, Al-Azhar University, Cairo, Egypt

[3]Department of Molecular Biochemistry and Pharmacology, Istituto di Ricerche Farmacologiche Mario Negri IRCCS, Via Mario Negri 2, 20156 Milano, Italy.

[4]Department of Soft Matter Nanotechnology, CIC Biomagune, Paseo Miramon 182, 20014 San Sebastian-Donostia, Spain

[5]INGM Imaging Facility, Istituto Nazionale Genetica Molecolare, Via Francesco Sforza 35, 20122 Milano, Italy

[6]Experimental Hepatology Unit, Division of Immunology, Transplantation and Infectious Diseases, IRCCS San Raffaele Scientific Institute, Via Olgettina 58, 20132 Milano, Italy.

[7]Irish Centre for Vascular Biology, School of Pharmacy and Biomolecular Sciences, Royal College of Surgeons in Ireland, Dublin, Ireland.

[8] Ludger Ltd., Culham Science Centre, Abingdon, Oxfordshire OX14 3EB, United Kingdom

[‡]Contributed equally as a first author.

*** Corresponding authors : marcomonopoli@rcsi.ie (Marco P. Monopoli) and paolo.bigini@marionegri.it (Paolo Bigini)**

Supplementary information

Table S1. Experimental conditions were used for the surface modifications. C_{NP}, $c_{DDA,}$ and $c_{PEG\ 2k}$ refer to the concentration of NPs, DDA, and PEG, respectively. $R_{p/Area}$ refers to the number of PMA monomers added per nm^2 of effective NP surface. A refers to the centrifugation acceleration (g = 9.81 m/s^2) and t refers to the centrifugation time.

Sample	Phase transfer		Polymer coating		
	c_{PEG2k}/c_{NP}	c_{DDA} [mmole]	$R_{P/Area}$ [nm^{-2}]	Centrifugation	
				a [g]	t [min]
**55-GNPs**	$1 \cdot 10^5$	60	3000	2570	30
**65-GNRs**				8330	

Table S2. Mean hydrodynamic diameters d_h in water as determined from the data shown in figure 1. $d_{h,N}$ is derived from the number distribution. $d_{h,I1}$ and $d_{h,I2}$ are derived from the 1st and (if existing) 2nd peak from the intensity distribution. $d_{h,Z}$ is derived from the Z-average, PDI is the polydispersity index and ζ is the ζ -potential of surface coating. Please notice that the size values for GNRs have no any physical meaning because they are not spherical NPs. It is intended only to show the colloidal stability and changes in the intensity distribution of d_h at the different steps of surface modifications.

Sample	Coating	$d_{h,N}$ [nm]	$d_{h,I1}$ [nm]	$d_{h,I2}$ [nm]	$d_{h,Z}$ [nm]	PDI	ζ[mV]
**55-GNPs**	CTAC	48.1 ± 0.8	66.4 ± 0.03		62.3 ± 0.3	0.04 ± 0.04	35.9 ± 1.1
	PMA	48.08 ± 1.2	72.08 ± 0.3		66.1 ± 0.3	0.07 ± 0.01	- 33.2± 1.49
	A2G2S2	56.8 ± 3.3	97.8 ± 0.7		88.7 ± 1.7	0.17 ± 0.01	-30.7 ± 0.25
	SNA	70.3 ± 42.9	175.3 ± 45.1		574.1±152.8	0.58 ± 0.15	-
**65-GNRs**	CTAB	3.23 ± 0.1	5.54 ± 0.1	70.76 ± 1.1	12.4 ± 0.14	0.52 ± 0.01	47.4 ± 1
	PMA	4.68 ± 0.1	7.36 ± 0.1	74.24 ± 1.3	11.8 ± 0.1	0.44 ± 0.01	- 36.2 ± 1.2
	A2G2S2	7.78 ± 0.3	12.9 ± 0.4	83.4 ± 0.7	20.7 ± 0.5	0.45 ± 0.01	-28.1 ± 0.43
	SNA	19.3 ± 10.4	33.4 ± 4.2	208.2 ± 3.3	176.3 ± 6.7	0.31 ± 0.02	-

Table S3. Shows the level of LPS in each particle sample.

Sample	Coating	EU/ml	Administrated Endotoxin EU/ injection does*
**55-GNPs**	PMA_55-HNPs	0.18	0.036
	A2G2S2_55-GNPs	0.21	0.042

**65-GNRs**	PMA_65-GNRs	0.40	0.08
	A2G2S2_65_GNRs	0.47	0.094

* The limit is stated as 5 endotoxin units (EU) per kg for intravenous injection, and it depends on the mouse's weight. Considering a mouse weight as an average of 0.03 kg, then the overall limit should be 0.15 EU (5 EU/kg*0.03kg).

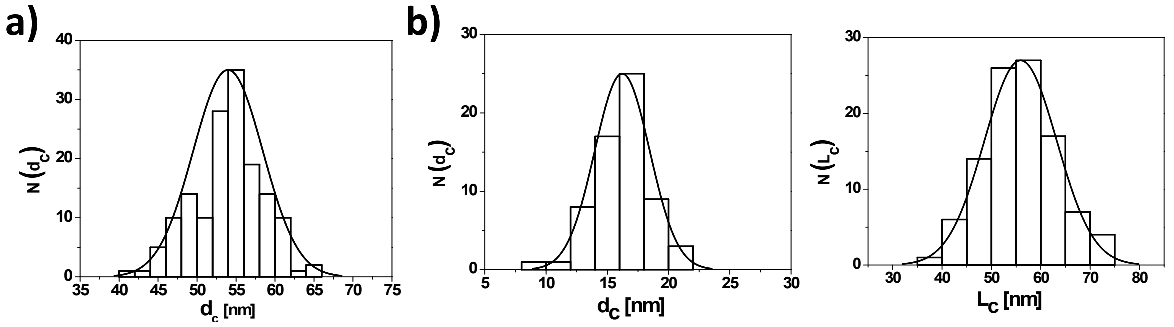

Figure S1. a) Size distribution histograms of 55-GNPs, plotted as number of NPs $N(d_c)$ that have a core diameter of dc= 54.3± 4.2 nm. b) Size distribution histograms of 65-GNRs, plotted as number of NPs $N(d_c)$ that have aa core diameter (histograms to the left) of d_c= 16.01 ± 2.9 nm and as number of NPs $N(L_c)$ that have a core length (histograms to the right) of L_c= 65.6 ± 9.4 nm.

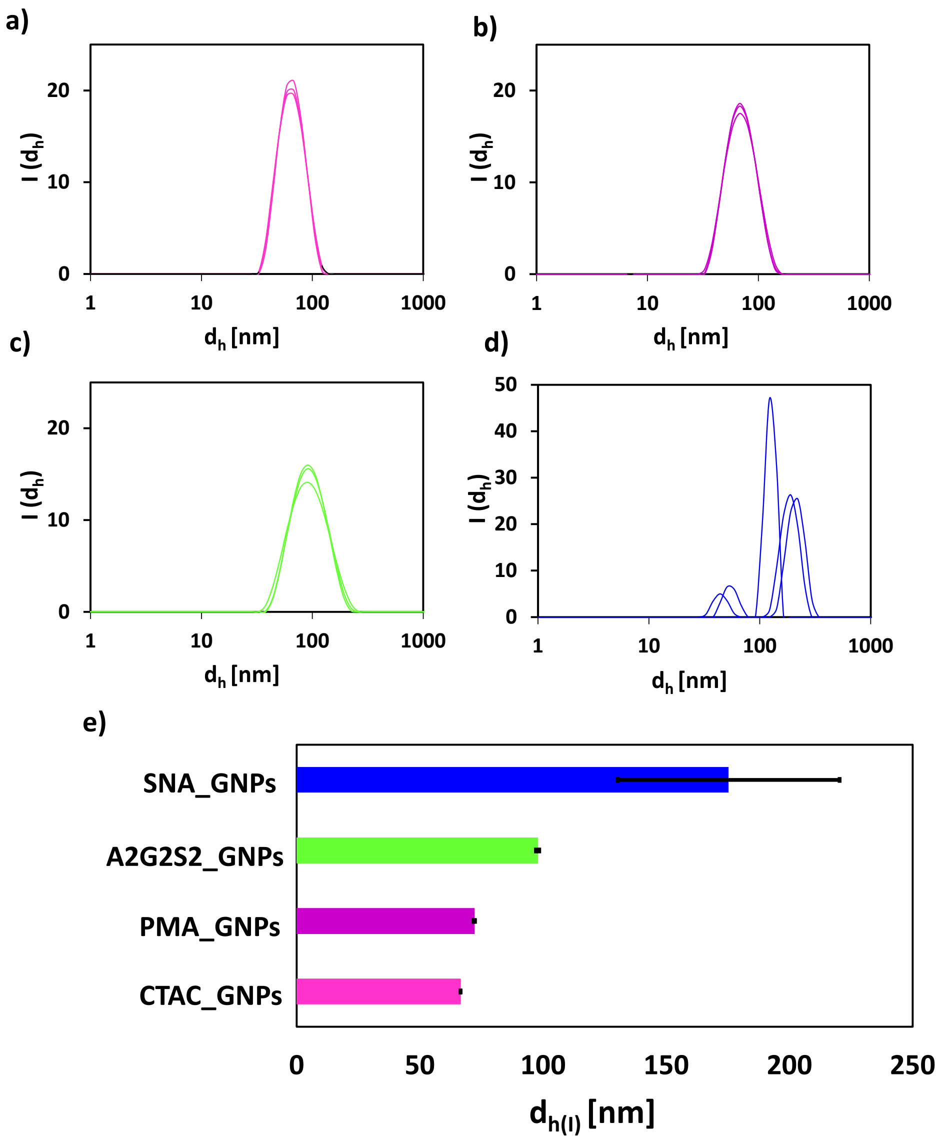

Figure S2. Intensity distribution I(d_h) of the hydrodynamic diameter d_h of 55-GNPs after a) CTAC-capping, b) PMA-coating, c) A2G2S2 conjugation and d) after interaction of A2G2S2 conjugated NPs with SNA. e) Mean hydrodynamic diameter $d_{h(I)}$ (± standard deviation) derived from the intensity distribution ($d_{h,(I)}$,Table 1) for GNRs after CTAC-capping (pink), PMA-coating (violet), glyco peptide conjugation (green) and after interaction of A2G2S2 conjugated NPs with SNA (blue).

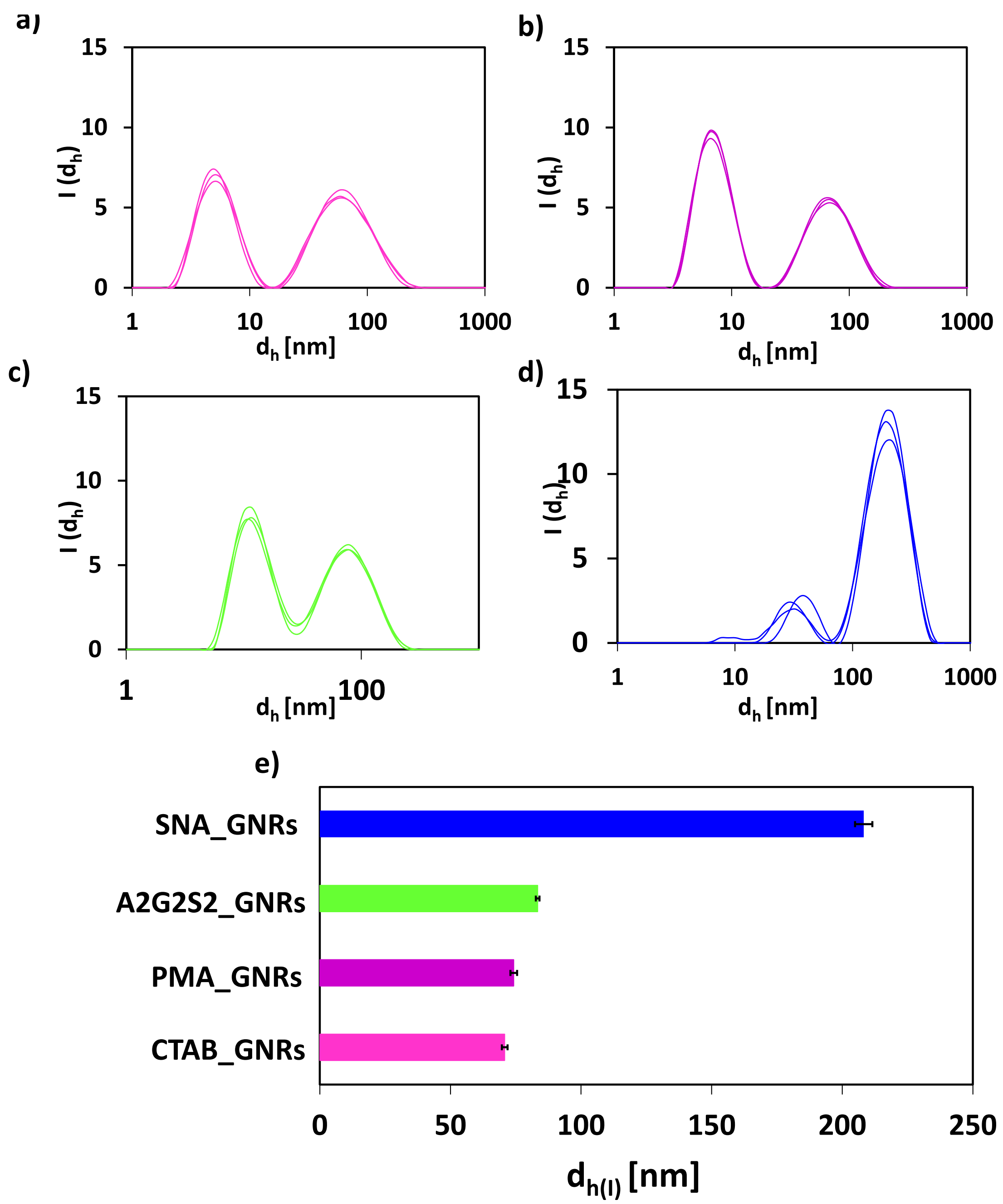

Figure S3. Intensity distribution I(d_h) of the hydrodynamic diameter d_h of 65-GNRs after a) CTAB-capping, b) PMA-coating, c) A2G2S2 conjugation and d) after interaction of A2G2S2 conjugated NPs with SNA. e) Mean hydrodynamic diameter $d_{h(I)}$ (± standard deviation) derived from the intensity distribution ($d_{h,I1}$,Table 1) for GNRs after CTAB-capping (pink), PMA-coating (violet), glyco peptide conjugation (green) and after interaction of A2G2S2 conjugated NPs with SNA (blue).

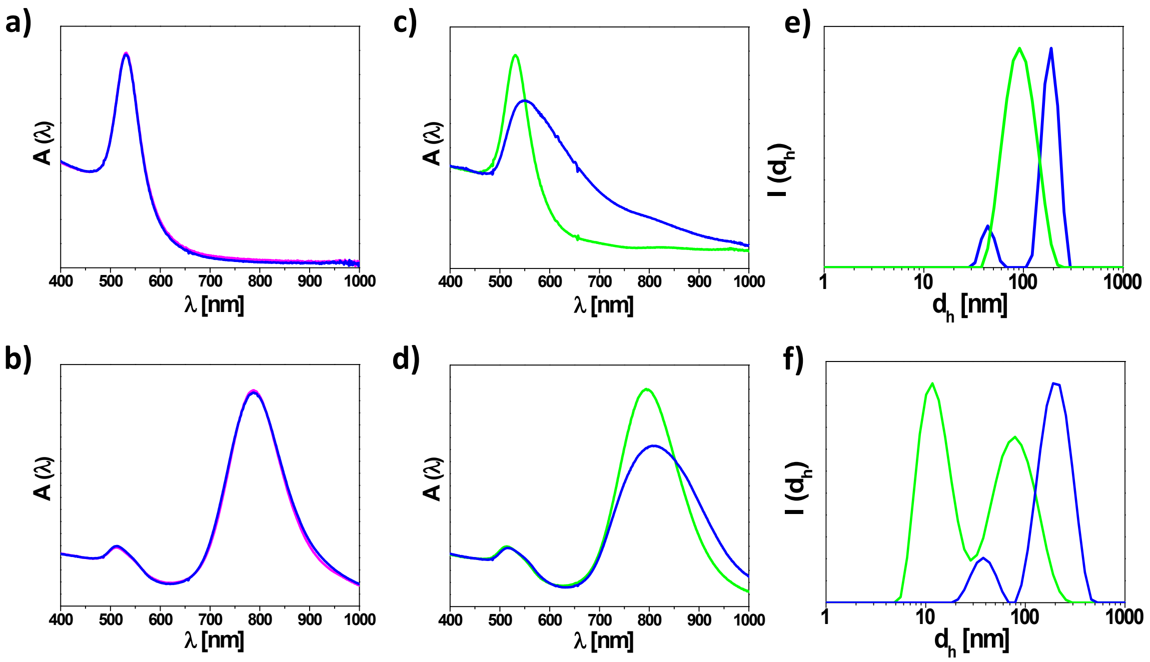

Figure S4. Lectin binding assays were conducted using GlycoNP-conjugates and SNA. a-b) The normalized absorption spectra of PMA-coated nanoparticles, a) 55-GNPs, and b) 65-GNRs, were measured both in the absence (pink) and presence (blue) of SNA. c-d) Normalized UV-vis absorbance spectra were recorded for both 55-GNPs (top) and 65-GNRs (bottom) after incubation in the absence and presence of SNA. e-f) Intensity distribution of hydrodynamic diameter (dh [nm]) for 55-GNPs (top) and 65-GNRs (bottom) was analyzed after incubation in the absence and presence of SNA.

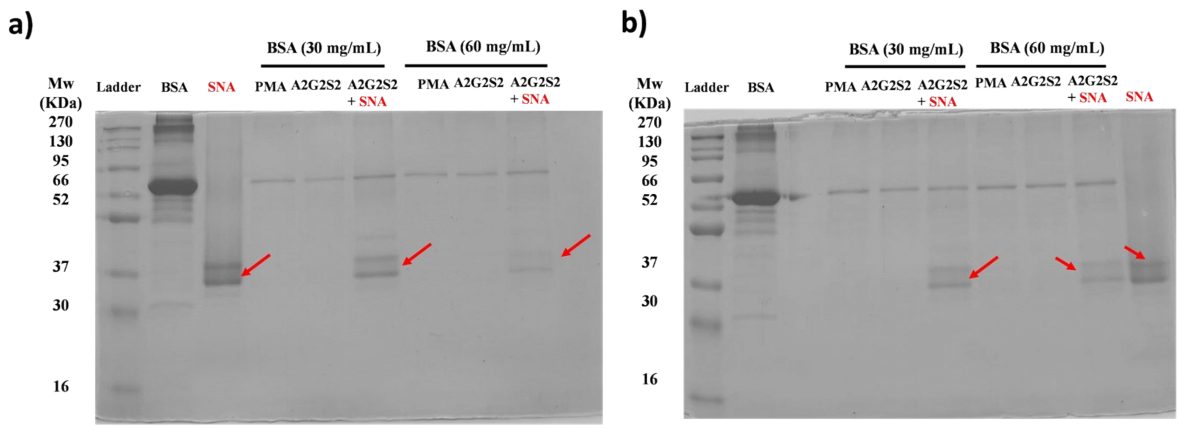

Figure S5. SDS-PAGE images of protein patterns adsorbed to PMA coated/ A2G2S2 conjugated a) 55-GNPs and b) 65-GNRs after exposure to protein-rich media (BSA). a & b) Proteins patterns of particles after exposure to 30 mg/mL and 60 mg/mL of BSA in absence of SNA (Lanes: PMA and A2G2S2) or presence of SNA (Lane: A2G2S2 + SNA) for 24 h. As a control, BSA and SNA samples were run through the gel.

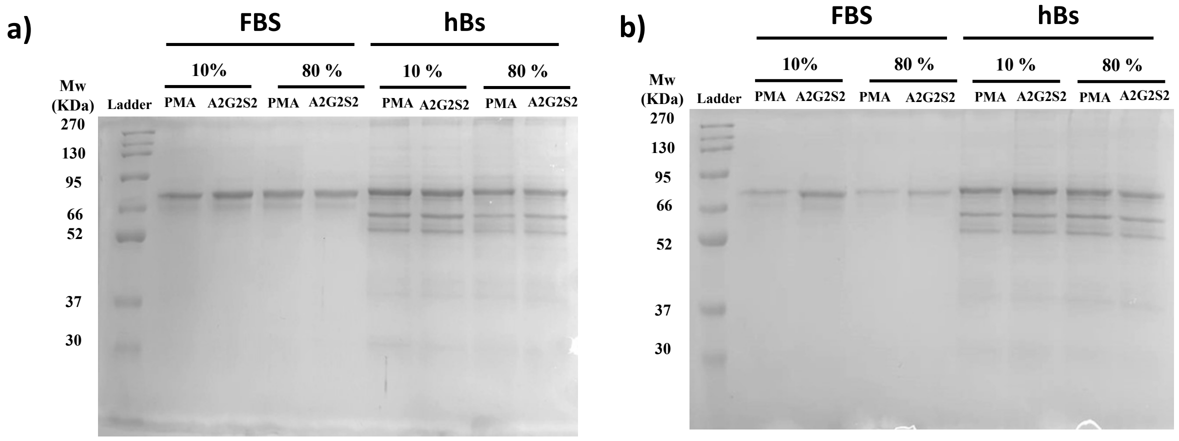

Figure S6. SDS-PAGE images of protein patterns adsorbed to PMA coated/ A2G2S2 conjugated a) 55-GNPs and b) 65-GNRs after exposure to different concentrations (10 % and 80 % of FBS, or hBs) at 37 ^{o}C for 1 h. In all gels, a standard protein ladder of different Mw was run through the gel to identify the Mw of proteins.

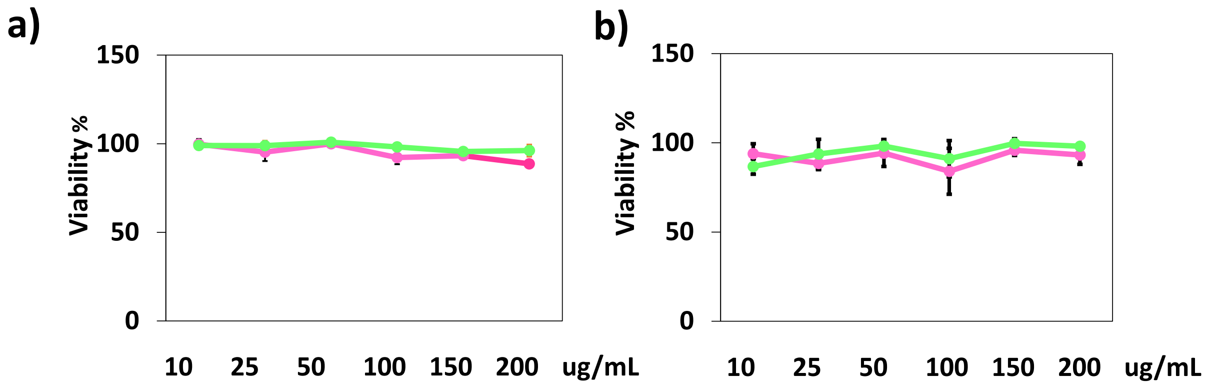

Figure S7. Cellular viability (%) as determined by the LDH assay with HepG2 cell lines exposed to a) 55-GNPs and b) 65-GNRs for 24 h at the indicated concentrations of NPs (µg/mL). Colour codes are assigned to PMA_55-G NPs/65-GNRs (pink) and A2G2S2_55-GNPs/65-GNRs (green). Data presented as mean ± SD from 3 wells per condition.

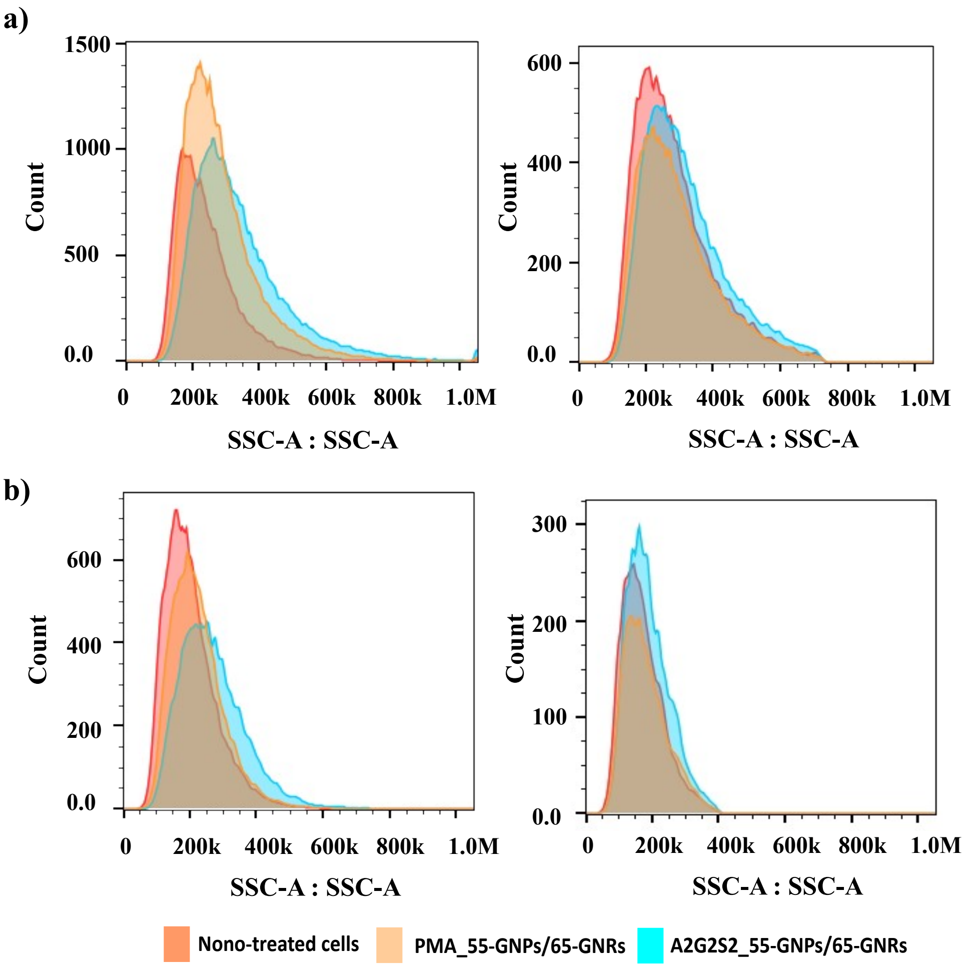

Figure S8. Flow cytometry-side scattering intensity (SSC-A) histograms of HepG2 cells treated with a) 55-GNPs and b) 65-GNRs in serum free (left) and 10% FBS (right) culture media for 4 h. Colour codes w are assigned to non-treated cells (dark orange), after treatment with PMA coated NPs (light orange), and A2G2S2 conjugated NPs (cyan).

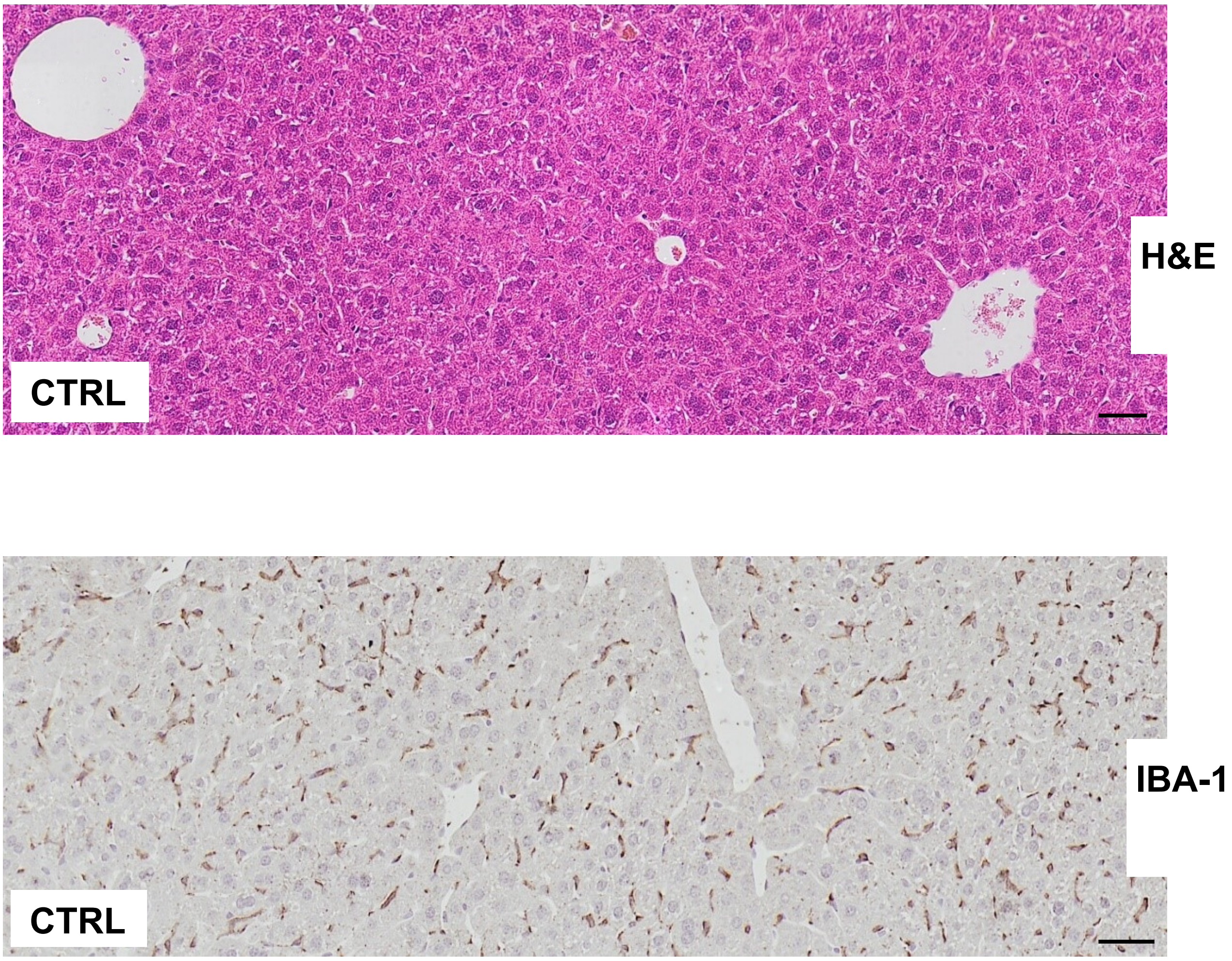

Figure S9. Histology of CTRL liver by H&E and IBA-1 staining. Representative micrograph of liver from non-treated mice. Scale bars = 100 μm.
